# Synthesis and Crystallization of Waterborne Thiol–ene Polymers: Toward Innovative Oxygen Barrier Coatings

**DOI:** 10.1021/acsapm.3c01128

**Published:** 2023-10-20

**Authors:** Justine Elgoyhen, Valentina Pirela, Alejandro J. Müller, Radmila Tomovska

**Affiliations:** † POLYMAT and Department of Applied Chemistry, Faculty of Chemistry, University of the Basque Country UPV/EHU, Avda Tolosa 72, 20018 Donostia-San Sebastián, Spain; ‡ POLYMAT and Department of Polymers and Advanced Materials: Physics Chemistry and Technology, Faculty of Chemistry, University of the Basque Country UPV/EHU, Paseo Manuel de Lardizábal 3, 20018 Donostia-San Sebastián, Spain; § IKERBASQUE, Basque Foundation for Science, Plaza Euskadi 5, 48009 Bilbao, Spain

**Keywords:** Thiol−ene sonopolymerization, thiol−ene miniemulsion polymerization, waterborne coating, self-nucleation, crystallization

## Abstract

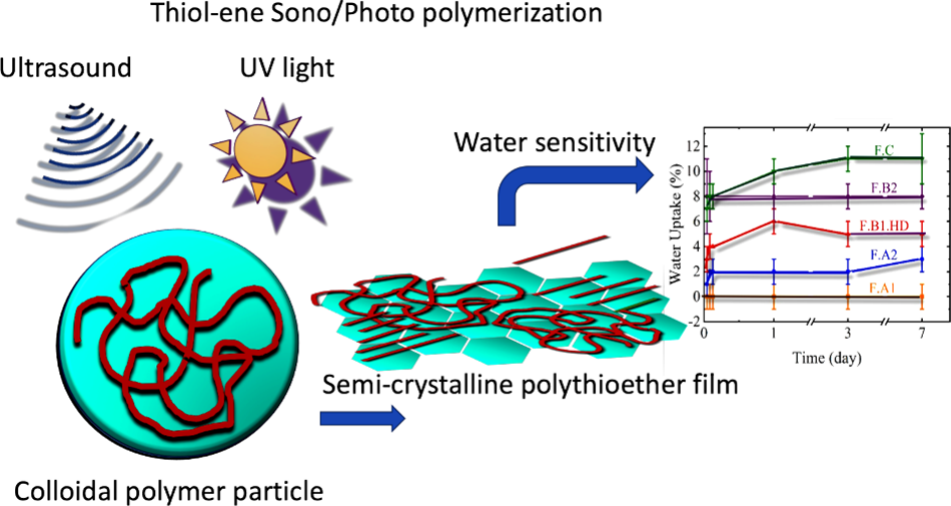

The synthesis of waterborne thiol–ene polymer dispersions is challenging due to the high reactivity of thiol monomers and the premature thiol–ene polymerization that leads to high irreproducibility. By turning this challenge into an advantage, a synthesis approach of high solid content film-forming waterborne poly(thioether) prepolymers is reported based on initiator-free step growth sonopolymerization. Copolymerization of bifunctional thiol and ene monomers diallyl terephthalate, glycol dimercaptoacetate, glycol dimercaptopropionate, and 2,2-(ethylenedioxy)diethanethiol gave rise to linear poly(thioether) functional chains with molar mass ranging between 7 and 23 kDa when synthesized at 30% solid content and between 1 and 9 kDa at increased solid content of 50%. To further increase the polymers’ molar mass, an additional photopolymerization step was performed in the presence of a water-soluble photoinitiator, i.e., lithium phenyl-2,4,6-trimethylbenzoylphosphinate, leading to high molar mass chains of up to 200 kDa, the highest reported so far for step grown poly(thioethers). The polymer dispersions presented good film-forming ability at room temperature, yielding semicrystalline films with a high potential for barrier coating applications. Nevertheless, affected by the polymer chemical repeating structure, which includes an aromatic ring, these thiol–ene chains can only crystallize very slowly from the molten state. Herein, for the first time, we present the successful implementation of a self-nucleation (SN) procedure for these types of poly(thioethers), which effectively accelerates their crystallization kinetics.

## Introduction

Oxygen and humidity barrier waterborne coatings for food packaging are a highly open market for innovations. Even though the current industrial barrier coatings based on waterborne poly(vinylidene chloride) (PVDC) present outstanding barrier properties,^1^ driven by the growing awareness of ecological issues, they should be replaced by halogen-free and more sustainable options. Pertaining to waterborne barrier coatings, the polymer microstructure is paramount for combining excellent barrier properties and good film-forming ability.^2^ Therefore, linear and crystallizable polymer chains embedded within a soft amorphous polymer matrix are indispensable. Because of that, the regular waterborne (meth)acrylic, alkyd, or polyurethane dispersions that lack crystallization potential are unsuitable for such applications. Thiol–ene chemistry and radical step growth polymerization in miniemulsion, initiated by irradiation (ultrasound and/or UV/vis light), appear as a suitable alternative for developing waterborne barrier coatings.

The feasibility of thiol–ene photopolymerization in aqueous dispersed media is well documented in the literature and has been performed in emulsion,^3,4^ miniemulsion,^5−11^ and suspension,^12^ giving rise to linear or cross-linked poly(thioether) dispersions in water, also called latexes. Among these processes, thiol–ene polymerization in miniemulsion is a privileged means to obtain high sulfur-content products with controlled colloidal properties and architecture. In this process, monomer droplets are formed in an aqueous continuous phase by a highly efficient homogenization device (such as a sonicator) and stabilized against diffusional degradation (Ostwald ripening) and droplet coagulation by using, respectively, a co-stabilizer and a surfactant. The key feature of miniemulsion polymerization compared to conventional emulsion polymerization is that no monomer transfer through the aqueous phase is required to make polymerization operative. Conversely, monomer droplets are directly nucleated to form polymer particles and become the loci of the reaction, acting as nanoreactors. This feature is particularly suited for thiol–ene step growth polymerization, in which a 1:1 ratio between the ene and thiol functionalities is essential to produce high molar mass (*M*_w_) polymers.

Noteworthy, Jasinski et al. reported the synthesis of linear poly(thioether ester) particles with film-forming ability by photopolymerization in miniemulsion.^7^ Linear chains based on the bifunctional monomers diallyl adipate (DAA) and ethylene glycol dithiol (EGDT) were synthesized with molar masses ranging from 35 to 57.5 kDa with a dispersity (*Đ*) of 2.5. The polymer presents a low glass transition temperature (*T*_g_) at −63 °C and yields a semicrystalline elastomeric film after water evaporation at room temperature. However, despite the low solid content (20%) of the final dispersions that lack practical significance, the authors acknowledged the limited utility of such film due to the low melting temperature of the crystals (18 °C) leading to poor mechanical properties. Introducing aromatic units in the polymer backbone is one of the strategies to stiffen significantly the final films obtained from thiol–ene aqueous dispersions. In another contribution, Jasinski et al. synthesized a film-forming poly(thioether) latex based on the monomers diallyl phthalate (DAP) and EGDT with a molar mass of 54.3 kDa and *Đ* of 2.7.^6^ Introducing a rigid aromatic ring into the polymer building block resulted in an increased *T*_g_ of −42 °C due to the less flexible chains and prevented the organization into crystalline domains.

Following these pioneering works related to thiol–ene waterborne dispersions, our attention was drawn on one hand toward high molar mass thiol–ene waterborne coatings containing aromatic rings in the polymer chain structure and their crystalline behavior and on the other toward high solid content aqueous dispersions, in the framework of intended industrial applications of barrier coatings, which are the main objectives of this work. To achieve those aims, three different couples of bifunctional thiol and ene monomers were selected for our study, in which diallyl terephthalate (DATP) was combined with either glycol dimercaptoacetate (GDMA), glycol dimercaptopropionate (GDMP), or 2,2-(ethylenedioxy)diethanethiol (EDDT). Polymer dispersions with solid content up to 50% were targeted, in which polymers with high molar mass were produced via step growth polymerization in miniemulsions.

One of the main drawbacks of thiol monomers is their limited shelf life, resulting in premature polymerization when mixed with ene monomers, also referred to as thiol–ene dark reactions.^13^ The uncontrolled nature of the thiol–ene dark reaction may impact experimental reproducibility, especially during the preparation of thiol–ene dispersions.^6^ The current strategies implemented to control thiol–ene reactivity consist of using radical scavengers and acidic compounds to prevent the radical or catalyst mediated thiol–ene addition reaction.^13^

Herein, we report the first attempt to take advantage of the high reactivity of thiol–ene systems. The benefit of a sonication step in miniemulsion polymerization was taken in the present work, and the thiol–ene mixtures were sonopolymerized simultaneously with the miniemulsification step, without the use of initiator. With this process, up to 50% solid content “prepolymers” were synthesized in aqueous dispersions. Ultrasound is a widely used tool in synthesis and has already been applied to efficiently initiate radical chain growth polymerization of methacrylate monomers^14−16^ and the coupling reaction of thiol–ene compounds in dispersed media.^17^ To the best of the authors’ knowledge, no study has been reported on thiol–ene sonopolymerization and even less in dispersed media.

In this work, to further enhance the polymers’ molar masses, a strategy of combining sonopolymerization and subsequent photopolymerization in the presence of a water-soluble photoinitiator was implemented. In this way, “prepolymers” based on the monomer couples DATP/GDMA, DATP/GDMP, and DATP/EDDT synthesized during the first step of sonopolymerization, were photopolymerized in the next step to produce up to 50% solid content film-forming poly(thioether) latexes with high molar masses. For the first time to the authors’ best knowledge, film-forming waterborne dispersions made of linear poly(thioether) polymers with large molar masses, suitable for the intended application in food packaging, were developed.

Crystallization has already been observed in linear poly(thioether) films.^6,7,18,19^ A controlled degree of crystallinity in the amorphous matrix films could yield smarter coatings, presenting a passive barrier to oxygen and water, and could be a good option to replace PVDC. Flexible and linear chains are more prone to form crystalline domains within the film, but on the other hand, some rigidity in the polymer chain is required to form a consistent film sufficiently good for coating applications. Coping with this contradiction, we investigated for the first time the crystallization behavior in linear poly(thioether) coatings. For that aim, we present here the implementation of a self-nucleation (SN) strategy^20,21^ to accelerate the crystallization kinetics of as-synthesized films, potentially improve the barrier properties, and add value to the films produced from thiol–ene chemistry. In this study, barrier properties were first estimated by water uptake experiments, and water permeation tests were performed for the films based on GDMA–DATP and GDMP–DATP.

## Experimental Section

### Materials

Diallyl terephthalate (DATP, >98%) and lithium phenyl (2,4,6-trimethylbenzoyl)phosphinate (TPO-Li, >99%) were purchased from TCI chemicals. 2,2-(Ethylenedioxy)diethanedithiol (EDDT, >95%), hexadecane (HD, >98%), and 2,2-azobis(2-methylpropionitrile) (AIBN, >98%) were purchased from Sigma-Aldrich. Glycol dimercaptoacetate (GDMA, >95%) and glycol dimercaptopropionate (GDMP, >95%) were kindly supplied by Bruno Bock. Tetrahydrofuran (THF, 99.9% HPLC grade) was purchased from Scharlab. Dimethyl sulfoxide-*d*_6_ (DMSO-*d*_6_, 99.5% D) and chloroform-*d* (CDCl_3_, 99.96% D) were bought from Eurisotop. Dowfax 2A1 was kindly supplied by Dow Chemicals. All chemicals were used as received without further purification, and deionized water was used as the dispersant media for the polymerization.

### Poly(thioether) Synthesis by Sonopolymerization and Photopolymerization in Dispersed Media

**Figure 1:**
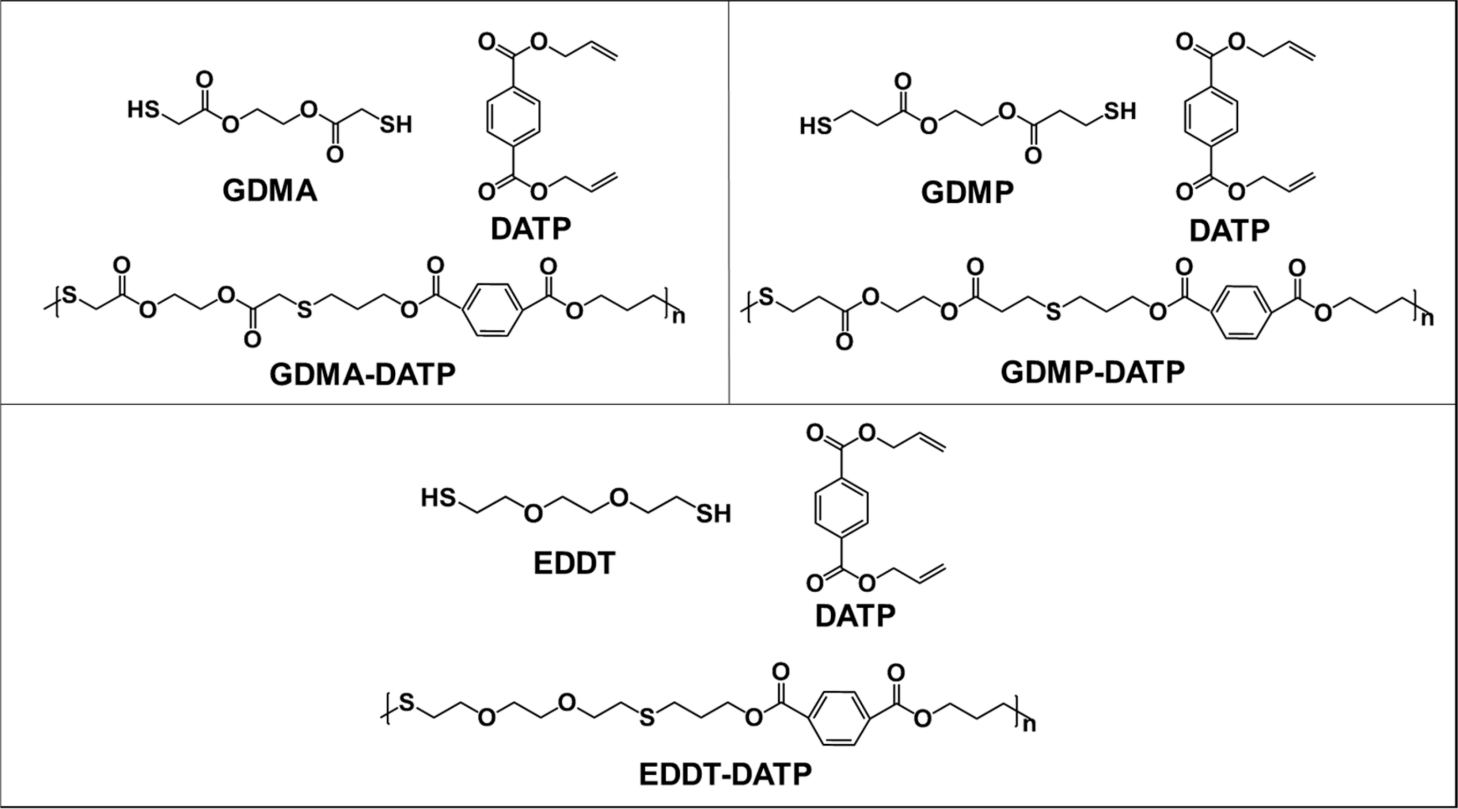
Chemical structure of the bifunctional monomers DATP, GDMA, GDMP, and EDDT, and poly(thioethers) GDMA–DATP, GDMP–DATP, and EDDT–DATP obtained from step growth polymerization.

In a typical reaction, the organic phase was prepared by mixing the bifunctional monomer pairs GDMA–DATP, GDMP–DATP, or EDDT–DATP (Figure 1), with a targeted 1:1 mol ratio of thiol–ene functionality, as depicted in Table S1, Supporting Information. Entries S.1–S.6 are basic formulations with 30% solid content. Entries S.1 and S.2 refer to sonopolymerization of the GDMA–DATP monomer couple in miniemulsion, whereas Entry S.3 stands for the same formulation in which 0.2 wt % (based on GDMA–DATP monomer mass) of the thermal initiator AIBN was added to the organic phase. In these formulations, no HD co-stabilizer was used because of the lack of solubility of HD in the monomer pair GDMA–DATP. Entries S.4 and S.5 are based on the EDDT–DATP monomer pair, with 6 wt % HD co-stabilizer and without HD, respectively. Entry S.6 refers to the GDMP–DATP monomer couple basic formulation (without initiator or co-stabilizer). Finally, entries S.7, S.8, and S.9 refer to 50% solid content basic formulations of each of the monomer couples studied GDMA–DATP, EDDT–DATP, GDMP–DATP, respectively. Both 30 wt % solid content and 50 wt % solid content dispersions were prepared by mixing the monomer pairs in an aqueous solution of surfactant (Dowfax 2A1, 1.5 wt %) for 5 min, followed by sonication in an ice bath to preform polymer particles. Sonication was performed using a Branson Digital sonifier at 70% amplitude, with a 0.5 s on and 1 s off pulsed program. Synthesis conditions and formulations are summarized in Table S1, Supporting Information.

For the subsequent photopolymerization step, 0.5 wt % (based on monomer mass) water-soluble photoinitiator TPO-Li was added after the sonication step to the relevant prepolymer dispersions. The reaction mixture was exposed to UV light for 1 h under stirring in a 630 mL quartz reactor placed in a UV chamber (model BS-03, Dr. Grobel UV-Elektronik GmbH) equipped with 20 mercury lamps with a maximum wavelength intensity at 368 nm and an irradiance of 7 mW/cm^2^.

### Film Formation

Various films with thickness ranging from 300 to 970 μm were prepared by casting the appropriate amount of the 30% and 50% solid content latexes into silicon molds. The films were dried at standard atmospheric conditions (25 °C and 55% relative humidity).

The films obtained after water evaporation of the 30 wt % solid content latexes are reported in Table 1. For clarity, films based on the GDMA–DATP monomer couple are denoted F.A, with F.A1 presenting a lower molar mass than F.A2. Films based on the EDDT–DATP monomer couple are denoted F.B and F.B1.HD displays lower molar masses than F.B2 and also contains 6 wt % HD. Finally, the film based on the GDMP–DATP monomers is referred to as film F.C. The main characteristics of the films are reported in Table 1.

**Table 1:** Films Obtained after Water Evaporation from the 30% Solid Content Latexes Based on GDMA–DATP, GDMP–DATP, and EDDT–DATP and Their Characterization in Terms of Molar Mass (*M*_w_), Glass Transition Temperature (*T*_g_), Degree of Crystallinity (*X*_c_), and Water Vapor Transmission Rate (WVTR)^a^

latex	film	monomers	synthetic process	HD (wt %)	*M*_w_ (kDa)	*T*_g_^b^ (°C)	*X*_c_^c^ (%)	WVTR^d^
S.1	F.A1	GDMA–DATP	S		9	–15.6	21	2
SP.2	F.A2	GDMA–DATP	SP		23	–7.8	15	
SP.4	F.B1.HD	EDDT–DATP	SP	6	52	–22.3	25	
SP.5	F.B2	EDDT–DATP	SP		81	–22.6	22	
SP.6	F.C	GDMP–DATP	SP		35	–14.6	20	12

a For the synthetic processes, S stands for sonopolymerization and SP stands for sonopolymerization combined with photopolymerization.

b *T*_g_ values were obtained from non-isothermal DSC analysis of the films.

c *X*_c_ values were obtained from the first heating scan of non-isothermal DSC analysis of the as-synthesized films and eq 2

d WVTR is in units of (g·mm)/(m^2^·day).

### Miniemulsion and Latex Characterization

The average particle size of the latexes and particle size distribution (PDI) were determined by dynamic light scattering (DLS) with a Zetasizer Nano ZS from Malvern Instrument. The samples for analysis were prepared by diluting a drop of the dispersion in approximately 1.5 mL of Milli-Q water to avoid multiple light scattering.

The colloidal stability of the prepolymer miniemulsion was assessed by measuring the backscattered signal over time with the Turbiscan equipment.

Gel Permeation Chromatography (GPC) with THF as the carrier was utilized to determine molar mass (*M*_w_) and molar mass distribution (*Đ*). Samples were prepared by dissolving dried polymers in THF at a concentration of 1 mg/mL. The GPC instrument consists of an injector, a pump (Waters 510), three columns in series (Styragel HR2, HR4, and HR6), and a differential refractometer detector (Waters 2410). The equipment was calibrated using polystyrene standards. The molar masses reported were obtained by comparison with the polystyrene standards.

Proton nuclear magnetic resonance (^1^H NMR) was used for the measurement of the final ene conversion or double bond conversion (DBC). NMR spectra were recorded on a Bruker 400 MHz at 25 °C. Samples were prepared by dissolving dried 20 mg aliquots in 0.5 mL of deuterated solvents CDCl_3_ or DMSO-*d*_6_.

### Film Characterization

The water uptake was measured by immersing films with similar rectangular shape (4.6 × 1.8 cm^2^) and thickness (∼0.6 mm) in distilled water in a closed vial. The films were then withdrawn, gently dried with paper, and weighed. The water uptake was determined as
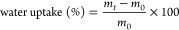
1where *m*_0_ is the weight of the film at *t*_0_ and *m*_*t*_ is the weight of the film at time *t*.

Thermogravimetric Analysis (TGA) of the films was performed with a PerkinElmer TGA 8000 Thermogravimetric analyzer. Samples were heated from 40 to 800 °C at 10 °C/min (purge gas: air at 40 mL/min).

The thermal properties and the crystallization of the samples were studied by Differential Scanning Calorimetry (DSC) employing a PerkinElmer DSC 8000 and an Intracooler II as a cooling system. Approximately 5 mg samples were encapsulated in aluminum pans. In the case of the film based on the EDDT–DATP monomer pair containing HD, a sealed aluminum pan was used.

**Figure 2:**
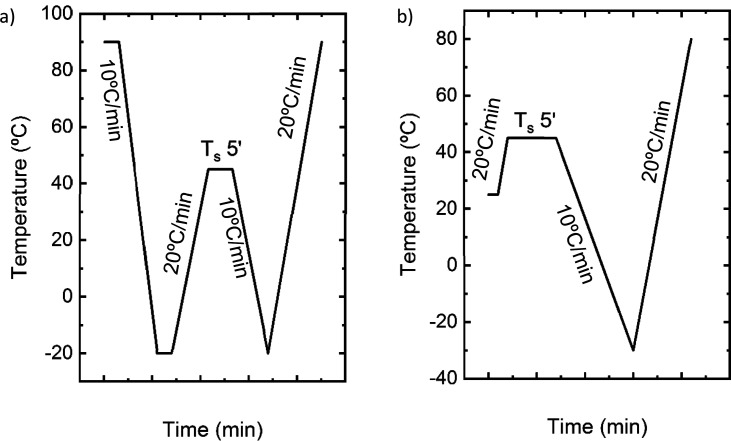
Schematic representation of the self-nucleation thermal protocol performed on (a) poly(thioether) films based on EDDT–DATP and containing HD (F.B1.HD) and (b) poly(thioether) films based on GDMA–DATP (F.A1, F.A2), EDDT–DATP (F.B2), and GDMP–DATP (F.C).

- Non-isothermal crystallization: For the non-isothermal procedure, samples were heated 30 °C above their melting point at 20 °C/min (that is, up to 80 °C for the F.A1 and F.A2 films, 90 °C for the F.B1.HD and F.B2 films, and 100 °C for the F.C film) and left 3 min in the molten state. They were then cooled at either 1 °C/min or 10 °C/min to −30 °C for the F.A1, F.A2, F.B1.HD, and F.B2 films and −40 °C for the F.C film. Films were subsequently heated at 20 °C/min to 80 °C (F.A1 and F.A2), 90 °C (F.B1.HD and F.B2) and 100 °C (F.C).
- Self-nucleation experiments: Self-nucleation (SN) is a thermal protocol in which self-seeds or self-nuclei are generated so that the nucleation density is greatly increased and therefore the overall crystallization kinetics can also be accelerated.^20−22^ For the SN experiments on F.A1, F.A2, F.B1.HD, F.B2, and F.C films, two thermal protocols were employed, as described here.
  - In the first procedure, see Figure 2a, which is the typical SN protocol, the F.B1.HD film was heated to 90 °C and held 3 min at this temperature to erase thermal history. The film was then cooled from its isotropic melt at 10 °C/min to −20 °C. In this step, a standard thermal history is imprinted in the sample, and the polymer crystallizes until saturation during cooling from the melt. The F.B1.HD sample was then heated up to a range of self-nucleation temperatures, denoted *T*_s_, and kept for 5 min at this temperature. During this thermal conditioning and depending on the *T*_s_ selected, the polymer can melt, self-nucleate, or self-nucleate and anneal. In the subsequent step, the F.B1.HD film is crystallized by cooling from *T*_s_ to −20 °C at 10 °C/min. A final heating scan was performed up to 90 °C at 20 °C/min.
  - In the second SN thermal protocol, see Figure 2b, applied to the F.A1, F.A2, F.B2, and F.C films, samples were directly heated up to a range of *T*_s_ temperatures at 20 °C/min, and kept 5 min at the chosen *T*_s_ temperature. *T*_s_ values were selected within the onset and the end of the melting peaks obtained in the first heating scan of the non-isothermal procedure. Films were then cooled at 10 °C/min to −30 °C (F.A1, F.A2, and F.C) and −20 °C (F.B2) and subsequently heated at 20 °C/min to 80 °C (F.A1 and F.A2), 90 °C (F.B2), and 100 °C (F.C).

The degree of crystallinity was calculated as follows:
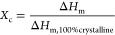
2As the melting enthalpies for the theoretically 100% crystalline polymers (Δ*H*_m,100%crystalline_) are not reported in the literature for the studied poly(thioethers), they were determined by using the group contribution theory of Van Krevelen.^23^ The values are reported in the Table S2 of the Supporting Information.

Water vapor transmission rate (WVTR) experiments of the F.A1 and F.C films were performed at 25 °C with a gravimetric cell containing a small amount of water. The gravimetric cell was sealed by the films F.A1 and F.C. The cell was put on a weighing scale with a readability of 10^–5^ g and the weight loss in time (Permeate flow) of the cell, solely due to the permeation of the water vapor through the film F.A1 and F.C, was registered by means of a computer connected to the scale. The water vapor transmission rate can be defined by
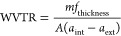
3where *m* is the weight loss in time (Permeate flow) of the cell, *f*_thickness_ is the film thickness (91.1 μm for F.A1 and 30 μm for F.C), *A* is the exposed area of the film (2.54 cm^2^), *a*_int_ is the water activity which is equal to 1 inside the cell, and *a*_ext_ is the penetrant activity outside the cell (assumed to be equal to the relative humidity in the case of water). In all cases, values of 0.3 of relative humidity (one percent) in the outside of the cell were considered.

## Results and Discussion

### Miniemulsion Polymerization of Thiol–Ene

In attempts to improve the reproducibility and the control of the thiol–ene polymerization process and to further increase the molar masses of the polymers, advantage was taken from the high reactivity of the thiol–ene system that usually results in premature polymerization and irreproducibility of the molar masses. Namely, instead of adding a radical scavenger to prevent the premature reaction during the sonication step employed to prepare the miniemulsion, this step was utilized to produce prepolymers that can be photopolymerized afterward to increase their molar masses.

For that aim, miniemulsions based on GDMA–DATP, GDMP–DATP, and EDDT–DATP were prepared and converted into “prepolymer” dispersions (S.*X*) during the homogenization step by sonication, without the use of radical scavenger or initiator. The reaction conditions and characteristics of the dispersions and polymers are reported in Table 2. Due to the interest in increasing the solid content for film forming aims, 30% and up to 50% solid content latexes were prepared. The effect of sonication time on the particle size and molar masses was studied for different thiol–ene pairs.

**Table 2:** Sonopolymers or Prepolymers Obtained by Sonopolymerization of the Bifunctional Monomers DATP, GDMA, EDDT, and GDMP^a^

entry	monomers	solid content (%)	sonication time (min)	particle size/PDI (nm)	DBC (%)	*M*_w_ (kDa)	*Đ*
S.1	GDMA–DATP	30	10	237/0.062	96	9	2.53
S.2	GDMA–DATP	30	10	194/0.042	96	13	3.18
S.3^b^	GDMA–DATP	30	10	202/0.18	97	11	2.83
S.4^c^	EDDT–DATP	30	7.5	175/0.103	99	23	3.65
S.5	EDDT–DATP	30	7.5	191/0.078	95	5	2.62
S.6	GDMP–DATP	30	7.5	274/0.214	89	7	2.47
S.7	EDDT–DATP	50	10	331/0.153	95	1	1.73
S.8	EDDT–DATP	50	12	198/0.165	95	6	2.93
S.9	GDMA–DATP	50	20	195/0.109	95	8	2.80
S.10	GDMP–DATP	50	10	213/0.127	96	9	2.85

a Sonication was performed at 70% of amplitude, with a 0.5 s on and 1 s off pulsed program, in the presence of 1.5 wt % of Dowfax 2A1 (calculated based on monomers mass).

b AIBN initiator, 0.2 wt % with respect to monomer mass, was used.

c HD co-stabilizer, 6 wt % with respect to monomer mass, was used.

Almost all sonopolymer latexes based on monomers GDMA and GDMP were synthesized without co-stabilizer, due to a solubility issue of the conventional co-stabilizer HD with the thiols. As the dithiol EDDT is miscible with HD, a miniemulsion based on the monomer pair EDDT–DATP was prepared using HD as co-stabilizer (entry S.4, Table 2). The same reaction was performed without HD (entry S.5, Table 2) for comparison aim with the other reactions and to study the effect of the co-stabilizer. The solid content of these latexes was 30%, and they were colloidally stable over time, as assessed by the repeatability of the light backscattered signal by the dispersions over time (Supporting Information, Figure S1) and by a particle size measured by DLS ranging from 175 to 331 nm with a narrow particle size distribution (PDI below 0.234) (Table 2). Synthesizing prepolymers during sonication (i.e., miniemulsification step) can be used as a strategy to obtain colloidally stable and reactive particles that can be further polymerized or functionalized in an additional step. Indeed, hydrophobic and high enough molar mass chains prevent monomer droplet diffusional degradation (Ostwald ripening), taking a role of co-stabilizers during miniemulsion/sonopolymerization. In thiol–ene polymerization, following a step growth mechanism, monomers are rapidly converted into dimers, trimers, and oligomers in very restrained time scales. These oligomers delay the Ostwald ripening effect because of a slower rate of diffusion through the aqueous phase.^15^ Combined with the surfactant preventing the droplet/particle degradation by coalescence, prepolymer particles, still reactive, were prepared.

The prepolymers or sonopolymers presented relatively high molar masses considering that they were polymerized by a step growth mechanism, achieving high conversion above 89% (DBC measured by ^1^H NMR). At 30% solid content, sonopolymers with a molar mass of about 9 kDa (entry S.1, Table 2) and 13 kDa (entry S.2, Table 2) were obtained for the GDMA–DATP monomer pairs with a *Đ* of 2.53 and 3.18, respectively. The different molar masses observed for the S.1 and S.2 sonopolymers can be explained by the difference in particle sizes (237 nm versus 194 nm, respectively), which is related to a radical confinement effect. In smaller particles, the number of radicals per particle is lower, resulting in less bimolecular termination and higher chain length of polymers.

Conversion was relatively high in all cases; nevertheless, there was still a high concentration of free functionalities. An attempt to increase the conversion and, subsequently, the molar mass of sonopolymers based on GDMA–DATP was carried out by adding 0.2 wt % (based on monomer mass) of the oil soluble thermal initiator AIBN (entry S.3, Table 2), as the sonication step induces an increase in temperature within the reactional mixture. Even though an ice bath is used to limit this effect, “hot spots” are generated in the reaction mixture in which very high temperatures can be reached, which likely thermally initiate the step growth polymerization and result in the synthesis of sonopolymers. In the presence of the thermal initiator AIBN, the conversion (DBC of 97%) and the final molar mass of S.3 sonopolymers (*M*_w_ of 11 kDa with a *Đ* of 2.83) was not increased significantly in comparison to the same reaction without initiator, S.1 (with a DBC of 96% and a molar mass of 9 kDa with a *Đ* of 2.53). Hence, the rest of the sonopolymers were synthesized without initiator addition. Even though in the presence of AIBN a higher number of radicals were created, this likely increased the bimolecular termination process (or recombination). Therefore, no positive effect on the molar mass was achieved.

For the EDDT–DATP monomer couple at 30% solid content with and without HD (entries S.4 and S.5, respectively, Table 2), polymers with a molar mass of about 23 kDa with a *Đ* of 3.65 and 5 kDa with *Đ* of 2.62 were obtained. The conversion reached in the presence of HD was much higher, with a DBC of 99% for S.4 and a DBC of 95% for S.5. The presence of HD provided improved colloidal stability and prevented monomer diffusion efficiently, which, as mentioned, is crucial to keep a 1:1 molar ratio of the thiol to ene within each droplet/particle to ensure large chain lengths. This is probably the main reason that the polymer synthesized with HD presents the highest DBC and molar mass compared with all other sonopolymers (with molar masses in the range of 5–13 kDa). This fact highlights that when the miniemulsion was prepared without HD, even though the oligomers are produced early in the reaction, there is still monomer diffusion that promotes the stoichiometric imbalance within individual particles (nanoreactors). Hence, the use of co-stabilizer seems to be an additional tool to increase the molar masses of the step grown emulsion polymers.

As the solid content is of utmost importance when speaking about the practical application of polymer dispersions, it was further increased to 50% (latexes S.7 to S.10, Table 2). The higher viscosity of 50% dispersions than 30% implies the requirement of higher energy input to achieve the same particle sizes and polymerization extent, and similar molar masses. To add additional energy, the sonication time was increased. Even at increased sonication time from 7.5 to 10 min, the poly(thioether) based on EDDT–DATP at 50% solid content (latex S.7) presents a significant drop in molar mass from 5 kDa at 30% (entry S.5, Table 2) to 1 kDa. Further augmentation of sonication time to 12 min likely provided sufficient energy to the 50% solid content dispersions to achieve similar conversion and molar mass as that at 30% (latexes S.8 and S.5, respectively). Increasing the sonication time for 50% solid content GDMA–DATP and GDMP–DATP from 10 to 20 min indeed resulted in similar conversions and molar masses as those for 30% solid content (S.9 versus S.1 and S.10 versus S.6).

Noteworthy, expanding sonication time even further led to coagulation. Indeed, higher sonication time can induce coagulation by increasing temperature and collision between droplets. Determining the optimal conditions for miniemulsification step can be challenging because the colloidal properties are also changed and affect the molar masses. On the one hand, droplet size decreases with sonication time,^24^ and the surfactant ensures the colloidal stability. On the other hand, too long sonication time provokes the collapse of monomer droplets, and the surfactant can no longer prevent coagulation. Herein, a good balance between these parameters was established in short sonication times, which seems indispensable for attaining stable oligomer dispersions.

### Photopolymerization

The oligomers produced by sonopolymerization contain free thiol and ene functionalities, which is the prerequisite to extend the molar mass in the second step of photopolymerization. For that aim, the prepolymer latexes S.2 and S.4 to S.11 were photopolymerized in an additional step in the presence of the water-soluble photoinitiator TPO-Li, yielding a range of photopolymers (SP, Table 3).

**Table 3:** Poly(thiolethers) Obtained from Sonopolymerization Combined with Photopolymerization Step^a^

			sonopolymer	photopolymer			
entry	monomers	solid content (%)	*M*_w_ (kDa)	particle size/PDI (nm)	DBC (%)	*M*_w_ (kDa)	*Đ*
SP.2	GDMA–DATP	30	13	185/0.06	99	23	4.02
SP.4^b^	EDDT–DATP	30	23	173/0.07	99	52	4.07
SP.5	EDDT–DATP	30	5	222/0.12	99	81	2.53
SP.6	GDMP–DATP	30	7	299/0.19	99	35	4.22
SP.7	EDDT–DATP	50	1	308/0.19	99	196	3.48
SP.8	EDDT–DATP	50	6	208/0.08	99	120	15.72
SP.9	GDMA–DATP	50	8	257/0.21	99	20	3.91
SP.10	GDMP–DATP	50	9	293/0.17	99	28	4.32

a Photopolymerization was performed for 1 h at an irradiance of 7 mW/cm^2^ in the presence of 0.5 wt % (with respect to monomers mass) TPO-Li.

b HD co-stabilizer, 6 wt % with respect to monomer mass, was used.

The average particle sizes of the latexes after photopolymerizations were not affected, implying colloidally stable dispersions and that only the free functionalities were reacting during this step within each particle. The conversions are substantially increased (DBC of 99%); hence the molar masses are increased. For 30% solid content dispersions, poly(thioethers) with average molar masses in a range of 23 kDa to 81 kDa were obtained with increased *Đ* ranging from 2.63 to 4.22 (entries SP.2, SP.4, SP.5, and SP.6, Table 3). During the photopolymerization process, the initiator radicals, created from the photoinitiator in combination with UVA light, react with the thiol-containing prepolymers produced during sonopolymerization (Figure S2, Supporting Information). The thiyl macroradical created reacts further with ene-containing prepolymer chains, and so on, producing much larger molar mass chains. The wider distribution of molar masses is likely a consequence of the bimolecular termination events between macroradicals with already produced chain lengths.

The same trend is observed for 50% solid content dispersions, giving rise to polymers with molar masses of 20 to 196 kDa (entries SP.7, SP.8, SP.9, and SP.10, Table 3), the last one being the highest reported for this type of polymer and polymerization process, to the best of the authors’ knowledge. The increase in the molar mass is probably a result of the high radical flux created after irradiation of the reaction mixture containing the photoinitiator that induced macroradical creation from each dead polymer chain that contained free thiol functionality. The high macroradical concentration within the small-particle nanoreactors finally resulted in a fast termination process leading to molar mass increase and, simultaneously, a wide distribution of chain lengths. In all cases, the largest kinetic chain length was obtained for EDDT–DATP based polymers (SP.4, SP.5, SP.7, and SP.8, Table 3). These results indicate that after sonopolymerization, the prepolymers based on the EDDT–DATP pair are still bifunctional. In the literature, the number-average molecular weight (*M*_n_) reported for poly(thioethers) containing EDDT monomers synthesized in miniemulsion are in a range of 20 and 55 kDa with the DAA^9^ or DAP^8^ dienes. Hence, the combination of sonopolymerization and photopolymerization turns out to be an efficient tool for producing particularly high molar mass step growth polymers.

It is worth mentioning that in the second step of photopolymerization, the presence of HD is not crucial, as the SP.4 in the presence of HD presented lower molar mass than SP.5 without the co-stabilizer. The reason behind this behavior is the very low concentration of free functionalities within the particles and the limiting effect of the monomer diffusion.

The synthetic approach combining sonopolymerization and photopolymerization seems particularly suited for obtaining thiol–ene step growth polymers with high molar masses. This could be explained by the very good colloidal stability obtained after the miniemulsification step for prepolymer particles P2 to P8, allowing further efficient photopolymerization, even at high solid content such as 50%.

### Film Formation and Crystallinity Study

Polymer microstructure is very important to combine excellent barrier properties and good film-forming ability; therefore, linear and crystallizable polymer chains are indispensable.

The latex film formation can be divided into three steps.^25^ After casting the latex on a substrate, the first step consists of water evaporation and particle ordering, followed by particle packaging, deformation, and coalescence in the second stage. The third and last step involves particle coalescence and subsequent interdiffusion of polymer chains across particle interfaces to fuse the particle boundaries. Interdiffusion of polymer chains occurs at temperatures above their *T*_g_ and is driven by Brownian motions. It is of primary importance regarding mechanical properties usually required for coating applications. Therefore, sufficiently low *T*_g_ (slightly lower than the film formation temperature) is required for proper chain interdiffusion. On the other hand, too low *T*_g_ values may result in a very soft polymer film that does not respond to the minimum requirements of mechanical resistance. These two opposed requirements are the main challenges of the film-forming ability of polymer dispersions.

Latexes based on the GDMA–DATP, EDDT–DATP and GDMP–DATP monomer pairs at standard atmospheric conditions (temperature of 25 °C and relative humidity of 55%) gave rise to the formation of homogeneous films F.A1 to F.C (Table 1 and photos in Figure S3, Supporting Information).

The prepared films are consistent and suitable for coating applications. Moreover, they are self-supported, which allows further characterizations. As the intended application is in food packing, the barrier to humidity or oxygen is of utmost importance. In the thiol–ene polymers, this is expected to be provided by the crystalline structures created within these films, as reported previously.^6,7^ The films based on EDDT–DATP (F.B1.HD, F.B2, F.B50, Table 1 and Figure S3, Supporting Information) and GDMP–DATP (F.C and F.C50, Table 1 and Figure S3, Supporting Information) are opaque, white, and ductile compared to the films based on GDMA–DATP (F.A1, F.A2, and F.A50, Table 1 and Figure S3, Supporting Information) that are transparent and flexible. This could be the first qualitative appreciation of the presence of crystalline domains, assuming that semicrystalline superstructural entities (such as spherulites) are larger than the wavelength of light, thereby causing the dispersion of light and hence opacity. In the following, a deeper study of the crystallization behavior of the thiol–ene polymeric materials prepared here is presented, with attempts to control their crystallization kinetics.

The thermal degradation curves of F.A1, F.A2, F.B1.HD, F.B2, and F.C films are shown in Figure S4, Supporting Information. Noticeably, all the studied thiol–ene films show degradation in the temperature range of 350–400 °C, which is at least 100 °C higher than the common (meth)acrylic waterborne polymers that degrade at temperatures ranging from 150 to 300 °C.^26,27^

The presence of aromatic functionality within the backbone can be one of the possible causes for the improved thermal resistance. The thermal stability was not affected by the molar mass of the chains, but it was affected by their chemical composition.

**Figure 3:**
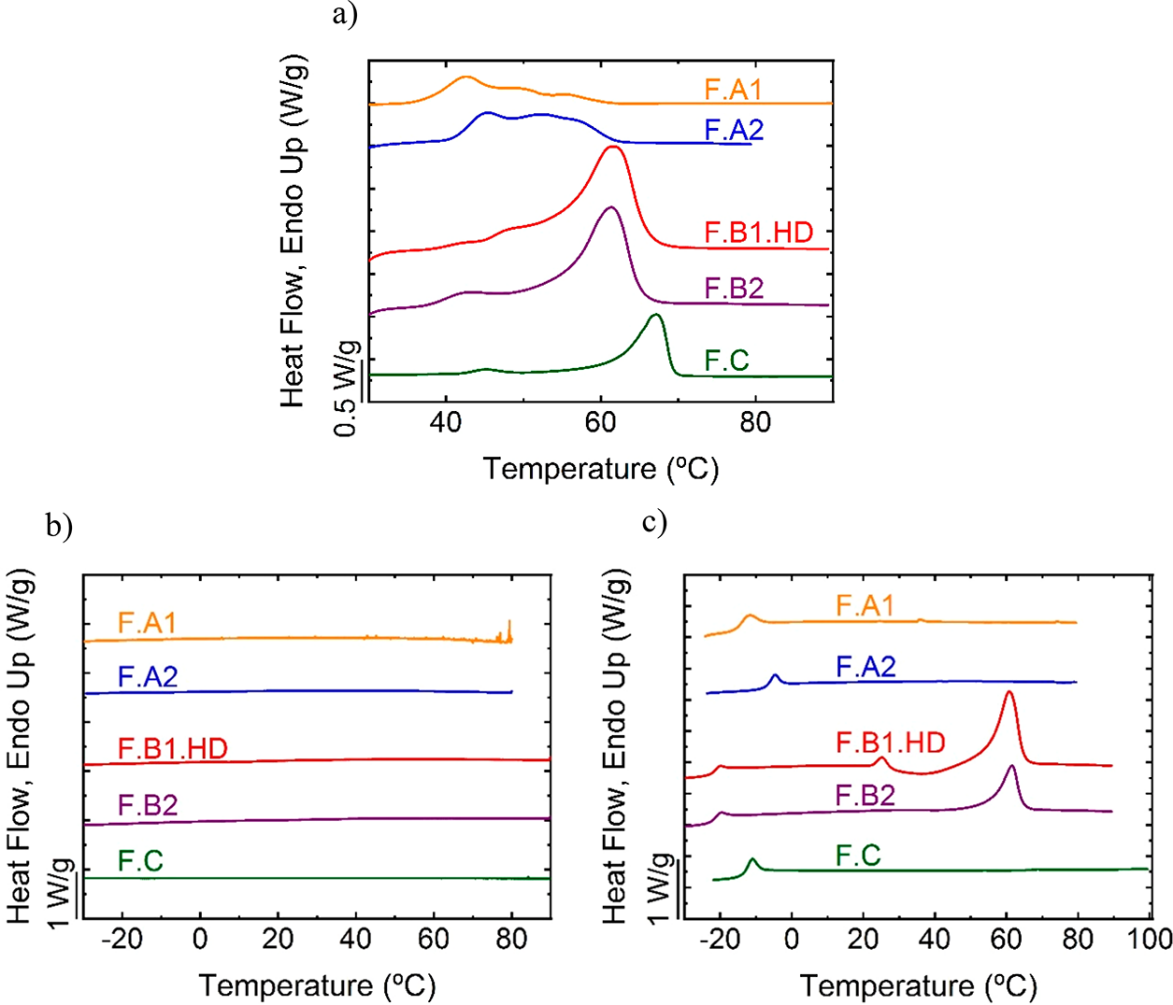
(a) First DSC heating scans at 20 °C/min of as-synthesized poly(thioether) films, (b) DSC cooling scans from the melt at 1 °C/min, and (c) subsequent heating scans at 20 °C/min of the F.A1, F.A2, F.B1.HD, F.B2, and F.C films.

The semicrystalline behavior of the as-synthesized films is evidenced by the presence of an endothermic melting peak in the first DSC heating scans performed on the samples, as shown in Figure 3a (the calorimetric values of interest obtained for this figure are listed in Table S3 of the Supporting Information).

The films F.A1 and F.A2, based on the same GDMA–DATP monomer combination, have a multimodal melting peak in the approximate temperature range of 40–60 °C (all melting peaks are reported in Table S3, Supporting Information), as shown in the first DSC heating scans (Figure 3a and Table S3, Supporting Information). The melting point is slightly shifted to higher temperatures for photopolymers with respect to the sonopolymer (F.A2 versus F.A1) due to the larger molar masses.

The F.B1.HD and F.B2 films based on EDDT–DATP have a melting peak at 62 °C (Figure 3a and Table S3, Supporting Information). In the F.B1.HD sample, a small endothermic peak is also observed at 48 °C, which is attributed to the melting of HD (see DSC scans in Figure S5, Supporting Information), which segregates from the polymer matrix in the film and crystallizes separately. The F.C film based on the GDMP–DATP monomer pair has a melting point of 67 °C, which is higher than those of the other films described previously.

The degree of crystallinity of each sample was determined from the first DSC heating scan, and the values reported in Table 1 were calculated using eq 2, see above.^23^

All samples present a similar degree of crystallinity of ∼20%, which is within the range required for barrier coating application.^28,29^ The F.A2 film, however, presents a slightly lower degree of crystallinity compared to F.A1. As both contain the same chemical structure, the decreased degree of crystallinity is attributed to the higher molar mass of F.A2. The crystallization kinetics of this higher molar mass polymer is probably slower.^30−32^

Non-isothermal crystallization experiments were performed on the films, and DSC traces are reported in Figure 3b,c.

When the films are slowly cooled from the molten state at 1 °C/min cooling rate, no crystallization peak is observed in the DSC scans (Figure 3b), demonstrating that these materials cannot crystallize from the melt, even when slowly cooled at 1 °C/min. They were able to crystallize during the film-casting process, as illustrated in their first DSC heating runs (Figure 3a). Once the polymer is molten and the crystalline history erased, they cannot crystallize from the melt at the minimum cooling rate employed (i.e., 1 °C/min). The presence of an aromatic ring in the repeating unit of the polymers coming from the DATP monomer (Figure 1) conveys rigidity to the chains and drastically slows their crystallization rate. These poly(thioethers) probably need much lower cooling rates (or longer times at specific temperatures) to allow the rearrangement of the polymer chains into crystalline domains. No melting peak is observed in the subsequent heating scan for the polymers F.A1, F.A2, and F.C based on GDMA–DATP and GDMP–DATP (Figure 3c) as no crystalline structures were formed during the cooling step.

Interestingly, the F.B1.HD and F.B2 films based on EDDT–DATP present cold crystallization during the heating scan and subsequent melting peaks at 61 °C (see Figure 3c), showing that these polymers can organize into crystalline domains when heated from the glassy state, after a previous cooling at 1 °C/min. It should be noted that these two materials have the lowest *T*_g_ values in Figure 3c, indicating that they are the most flexible among the three types of polymers synthesized. This is probably why they can exhibit crystallization upon heating from the glassy state. It should also be mentioned that polymers tend to nucleate when they are vitrified upon cooling. The ability to nucleate during vitrification can be exploited to observe crystallization from the glassy state in polymers that are not able to crystallize during cooling from the melt at specific cooling rates (e.g., this is commonly observed in poly(ethylene terephthalate)^33^ and poly(lactide)^34^).

For the film F.B1.HD, crystallization occurs upon heating from the glassy state, as shown by the exothermic crystallization peak at around 40 °C in Figure 3c. This phenomenon is called cold crystallization, and here it does not allow the full recovery of the original crystallinity the sample achieved during film formation, as Δ*H*_m_ in the second heating scan is substantially lower than the Δ*H*_m_ of the as-synthesized film (Table S3, Supporting Information).

F.B2 film behaves similarly, presenting lower Δ*H*_m_ in the second heating scan than in the as-synthesized film (Table S3, Supporting Information). In the case of film F.B2, the cold crystallization peak could not be clearly observed by DSC, which can be attributed to a slower cold crystallization during the scan (a fact that may make difficult the observation of cold crystallization, as it can be masked by the baseline), than its counterpart F.B1.HD. Film F.B1.HD contains 6 wt % of HD, which could act as a plasticizer or nucleating agent and slightly increase crystallization kinetics in comparison with F.B2. The non-isothermal DSC analysis of HD is presented in Figure S5 in the Supporting Information, and it shows that HD crystallizes upon cooling at 20 °C and melts at 30 °C. Hence, in the heating scan of film F.B1.HD in Figure 3c, HD is in its semicrystalline form at the onset of the cold crystallization exotherm, occurring around 24 °C, which means it could nucleate the polymer and accelerate the EDDT–DATP crystallization kinetics. Above 30 °C, HD is molten and can act as a plasticizer. Besides the presence of HD that induces slight differences in the crystallization behavior of the two films, F.B1.HD has a substantially lower molar mass than F.B2 (Table 1), which could also explain the faster crystallization kinetics for the latter.

**Figure 4:**
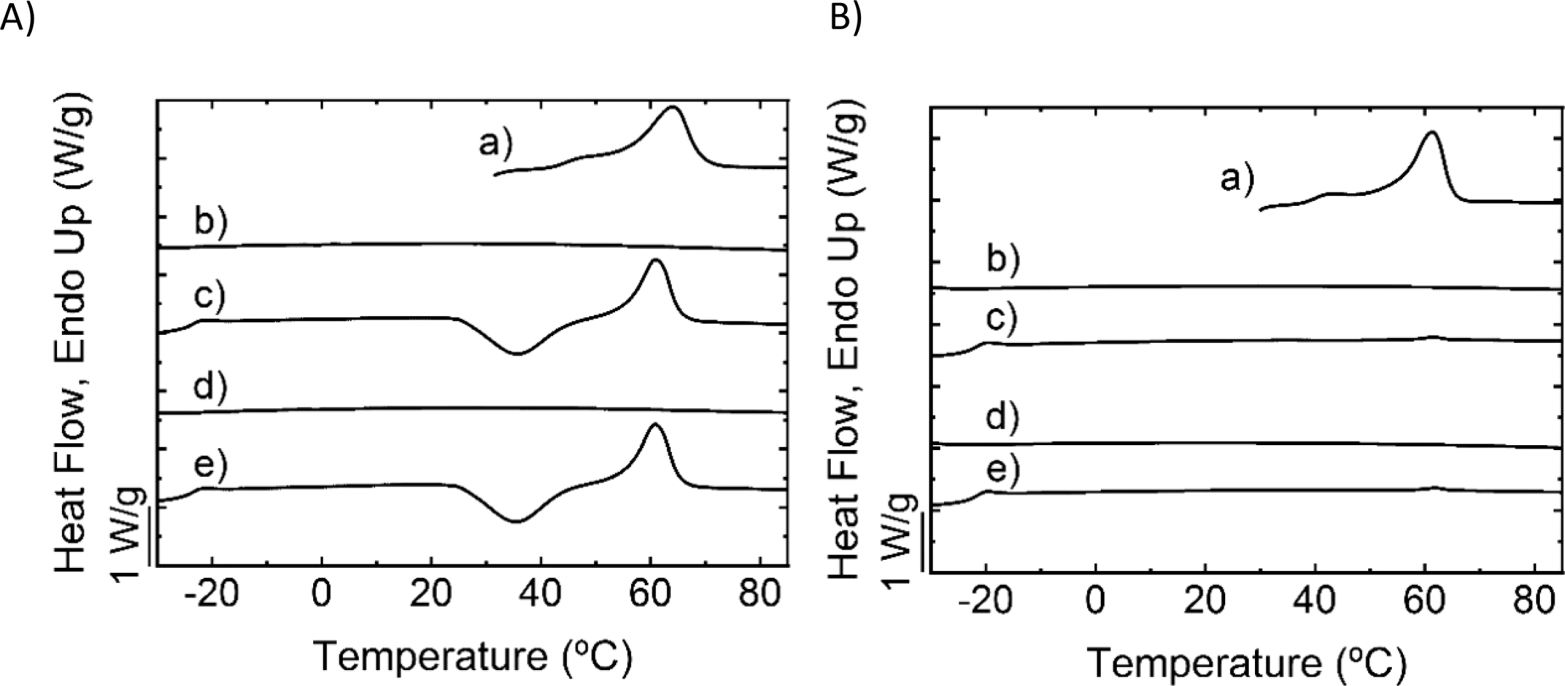
For (A) F.B1.HD film and (B) F.B2 film: (a) first DSC heating scan at 20 °C/min, (b, d) DSC cooling scans from the molten state at 10 °C/min and (c, d) subsequent heating scans at 20 °C/min.

Additional analysis of F.B1.HD and F.B2 films was performed by non-isothermal experiments in which the cooling rate was increased to 10 °C/min (Figure 4).

As depicted in Figure 4, cold crystallization and subsequent melting are observed for the F.B1.HD film after a cooling scan at 10 °C/min, whereas no melting peak is observed for the F.B2 film. The latter result shows that in the case of the F.B2, the occurrence of cold crystallization is dependent on the cooling rate. It is postulated that during the cooling rate at 1 °C/min (Figure 3), nuclei are formed during vitrification and promote the latter organization into crystalline regions during the subsequent heating scan. When the cooling rate is increased to 10 °C/min, the nuclei are not formed (i.e., the nucleation rate is too slow in this case), and cold crystallization is precluded. For the film F.B1.HD, which displays lower molar masses (Table 1), nuclei could be formed at cooling rates of 1 °C/min and 10 °C/min, as lower molar masses promote faster nucleation and cold crystallization.

All films displayed low *T*_g_ values, below 0 °C (Figure 4c and Table 1 and S3, Supporting Information), indicating that soft rubbery films can be produced at room temperature despite the rigidity induced by the aromatic rings in the polymer chains, which is counterbalanced by the aliphatic parts. The polymer molar mass affects the *T*_g_; thus, F.A1 with a lower molar mass (Table 1) than its counterpart F.A2 is softer, which explains a *T*_g_ decrease from −7.8 °C to −15.6 °C. A *T*_g_ lower than the film formation temperature ensures good cohesion of the obtained films. The mobile chains in the rubbery state interdiffuse and create entanglements efficiently in the last stage of the film formation process, which, together with the crystalline regions, yield tough, rubbery materials at room temperature. The supplement of rigidity brought by the aromatic ring of DATP, along with the presence of crystalline structures, increases the stiffness of the final material and promotes the formation of coherent coating films. As emphasized in the introduction, thiol–ene film-forming polymers that have been studied in previous works suffered from very low melting points, which restrained their utility, because the crystalline domains are melted at room temperature.^7^ Herein, the selected poly(thioethers) present significantly higher melting points than room temperature. The presence of crystalline domains within the films at room temperature could add value to the final properties of the materials, as crystallinity influences mechanical properties, thermal stability, and, most importantly for the present work, the barrier properties.

As the non-isothermal crystallization experiments on the films emphasize, the selected poly(thioethers) are slow-crystallizing material when they are cooled from the melt. One way to accelerate their crystallization kinetics is to implement a self-nucleation (SN) strategy, a thermal protocol in which self-nuclei are generated within the material at selected self-nucleation temperatures, *T*_s_.^21^ SN allows control of the nucleation step preceding the crystal growth. Nucleation is accelerated when polymer chains nucleate on pre-existing surfaces. In the case that polymer chains nucleate on preexisting crystals, the self-nucleation is very efficient, as the chains can epitaxially nucleate on their own existing crystal structures. SN exponentially increases the nucleation density, and thus the overall crystallization kinetics is accelerated, as it comprises both nucleation and growth.^35^

The conventional SN thermal protocol (Figure 2a) could be applied to the F.B1.HD film, as non-isothermal experiments have shown that this film could crystallize from the glassy state by cold crystallization, attesting to its faster crystallization kinetics than F.A1, F.A2, F.B2, and F.C films.

**Figure 5:**
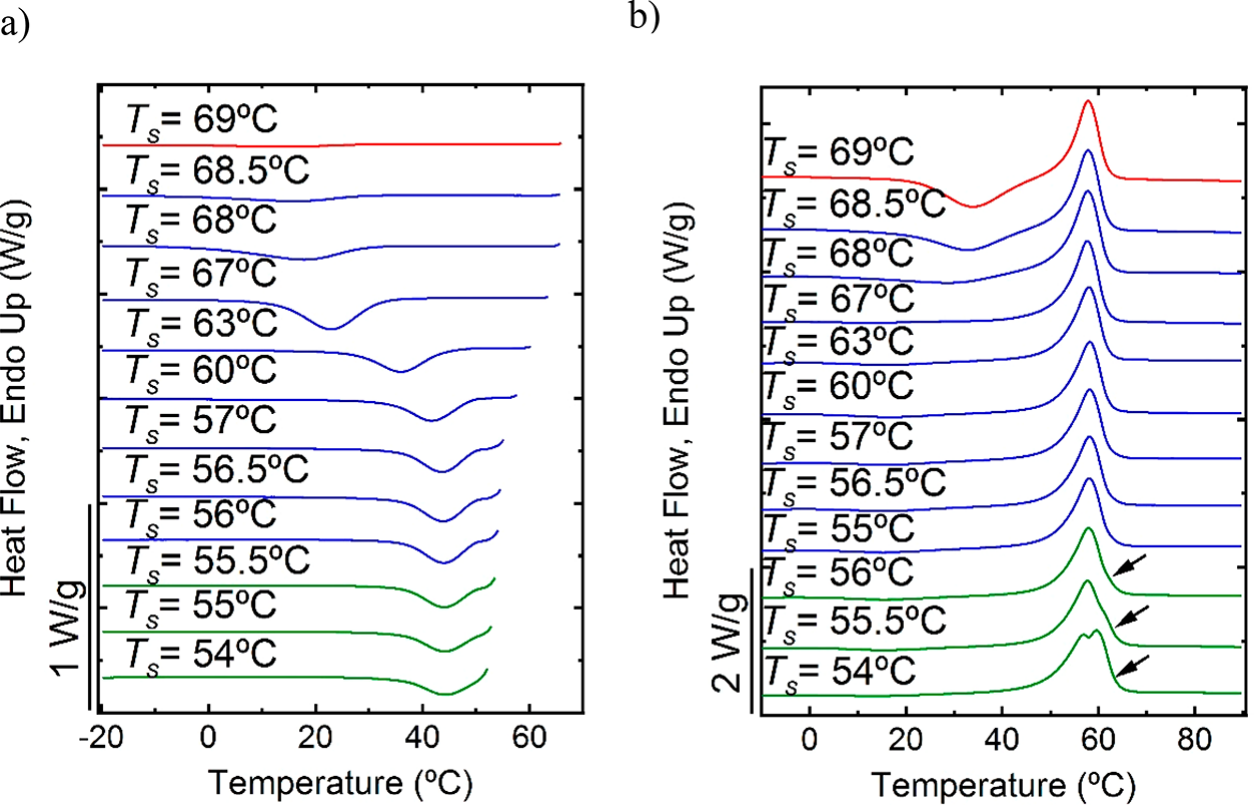
Self-nucleation experiments for F.B1.HD poly(thioether) film: (a) DSC cooling scans from the indicated *T*_s_ temperatures at 10 °C/min and (b) subsequent heating scans at 20 °C/min. Arrows show traces of annealing for the *T*_s_ measured within *Domain III*. The DSC curves have been drawn in different colors to represent the state of the sample in the different SN domains: red for *Domain I*, blue for *Domain II*, and green for *Domain III*.

For the F.B1.HD film, successful SN experiments were performed, and its overall crystallization kinetics was accelerated. In fact, after SN, exothermic crystallization peaks could be observed during cooling from selected *T*_s_ temperatures at 10 °C/min (see Figure 5a).

From the SN experiments, three self-nucleation domains can be defined as described by Fillon et al.^20^ In *Domain I* (or the melting domain), which occurs for *T*_s_ values well above *T*_m_, the polymer is completely molten. The thermal history and memory effect induced by previous crystallization are erased, and the crystallization temperatures (*T*_c_) obtained upon cooling are constant. According to the DSC traces in Figure 7a, F.B1.HD is in *Domain I* for *T*_s_ values equal to or higher than 69 °C. The exothermic peak upon cooling is not visible by DSC, as the absence of self-nuclei could not speed up crystallization kinetics during cooling from the melt. The sample crystallizes upon heating from the glassy state (cold crystallization exotherm visible in Figure 5b). So, in this case, the SN behavior differs from that reported for polymers that can achieve a standard state by crystallizing from the isotropic melt during a controlled cooling from the melt.^20−22^ Therefore, in the present case, the sample in *Domain I* exhibits the same behavior as in the non-isothermal experiments shown in Figure 5a.

**Figure 6:**
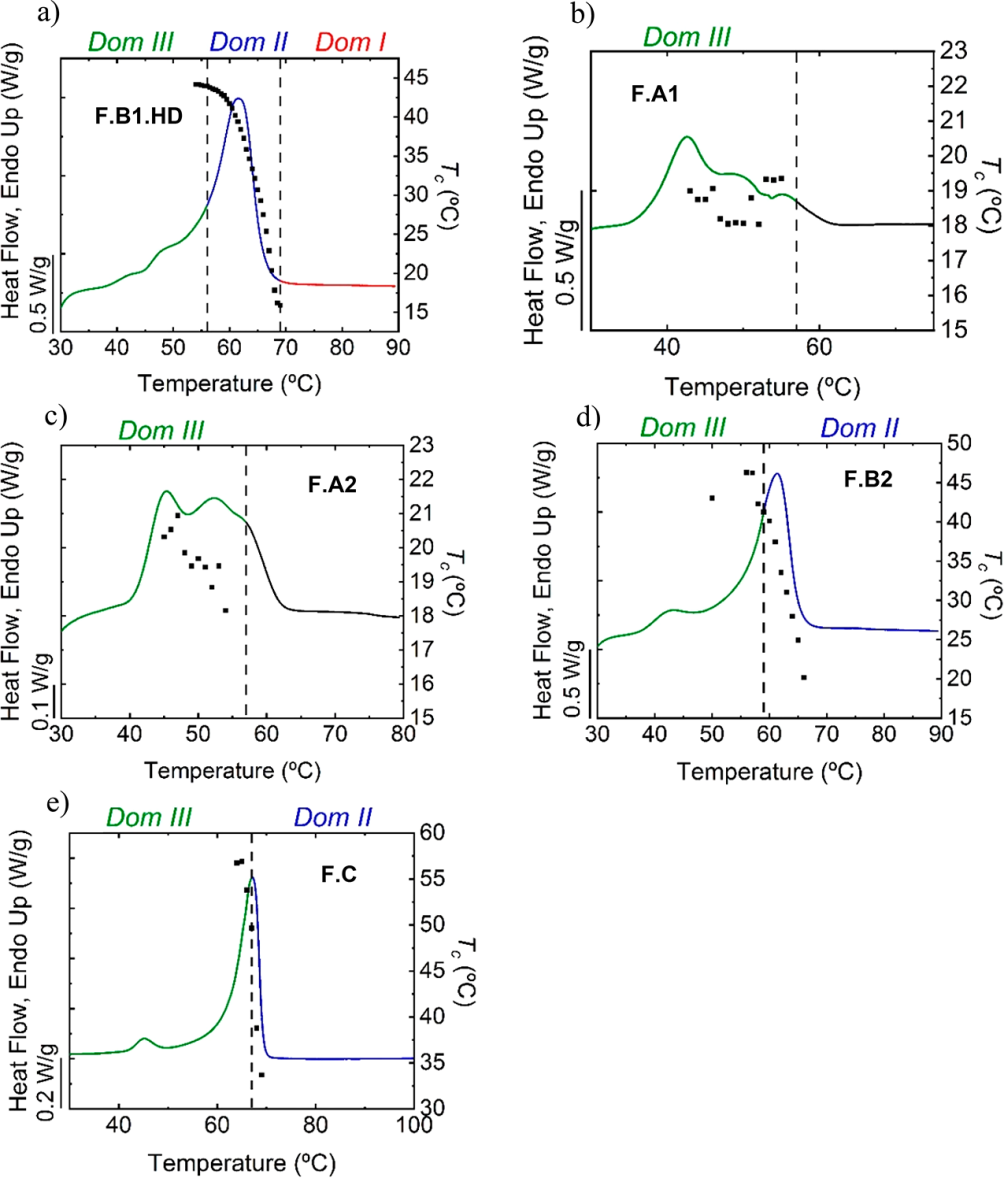
Peak crystallization temperature (black data points, plotted on the right-hand side *y*-axis) as a function of *T*_s_ (plotted on the *x*-axis) for (a) F.B1.HD, (b) F.A1, (c) F.A2, (d) F.B2, and (e) F.C superimposed on its first DSC melting endotherm. Values are taken from the self-nucleation experiments reported in Figure 5, and Figures S6, S7, S8 and S9 in the Supporting Information and the first heating scan of the film from Figure 3a.

In *Domain II* (or the exclusive self-nucleation domain), *T*_s_ values are high enough to melt most crystals and low enough to create self-nuclei. In the present case, the self-nuclei are composed of crystal fragments (i.e., self-seeds) that were not melted at *T*_s_, as indicated by the fact that the *T*_s_ temperatures are always within the melting range of the polymer (see Figure 6a). Therefore, according to previous recent literature, *Domain II*, in this case, can be classified as a self-seeding domain or *Domain IIb*, as *Domain IIa* or the melt memory domain is absent in this case because self-nuclei cannot be created at temperatures where all the crystals in the samples have been molten (i.e., at temperatures higher than the end melt temperature, i.e., at temperatures where the DSC cannot register any more latent enthalpy of fusion). The interested reader is referred to ref (22).

Self-seeds constitute ideal nucleation sites for epitaxially crystallizing the polymer. They increase the nucleation density greatly, inducing a shift of the crystallization peak from the melt to higher temperatures.^20−22,36^ According to DSC traces in Figure 5a, the F.B1.HD film is in *Domain II*, or the self-nucleation domain, in the range of *T*_s_ from 56 to 68.5 °C: the overall crystallization kinetics is accelerated, with the crystallization peak visible in the DSC traces upon cooling and clearly shifting toward higher temperature values, while increasing its magnitude at the same time (see Figure 6).

In Figure 6a, the *T*_c_ of F.B1.HD is presented as a function of *T*_s_ temperature superposed on the first heating scan of the film. Please note that for the DSC scan, the *x*-axis represents the experimental temperature during the scan, while for the plot of *T*_c_ versus *T*_s_, the *x*-axis represents the *T*_s_ temperatures employed during the SN treatment. The plot of *T*_c_ against *T*_s_ shows a clear increase in *T*_c_ as *T*_s_ decreases in the *Domain II* temperature range. Nevertheless, the range of *T*_s_ is still high enough to avoid any annealing of the pre-existing crystals (or self-seeds), which is the main difference with *Domain III*.

In *Domain III*, or the self-nucleation and annealing domain, *T*_s_ values are lower than in *Domain II*, so the material is only partially melted. The unmolten crystals experience an annealing process during the 5 min holding time at *T*_s_. Therefore, *Domain III* can be differentiated from *Domain II* by the presence of annealing traces in the final DSC heating scan of the experiment. Annealing of the unmolten crystals is visible in the heating scans by DSC (the melting of the annealed crystal population is signaled by arrows in Figure 5b), as annealed crystals melt at higher temperatures. The F.B1.HD film is in *Domain III* at *T*_s_ lower than 55.5 °C. In Figure 6a, the vertical lines divide the temperature range into the three different domains for the F.B1.HD film.

The thermal protocol had to be adapted for the other samples, F.A1, F.A2, F.B2, and F.C, as these poly(thioethers) do not recrystallize from the molten or the glassy state. The step in which films are cooled from the isotropic melt to create a standard thermal history and polymers crystallize until saturation is omitted. As presented in Figure 2b, the as-synthesized films are directly heated to a range of *T*_s_ values and conditioned for 5 min at these temperatures. *T*_s_ values were selected within the onset and the end of the melting peak obtained from the first DSC heating scan of the non-isothermal procedure (Figure 3). With this thermal protocol, it is expected that polymer samples from F.A1, F.A2, F.B2, and F.C films are in the SN *Domain II* or *III*.

Successful SN experiments were performed for F.A1, F.A2, F.B2, and F.C, and the overall crystallization kinetics were accelerated, as exothermic crystallization peaks can be observed by DSC upon cooling at 10 °C/min from a range of *T*_s_ values (Figures S6a, S7a, S8a, S9a, Supporting Information). Considering the thermal treatment, crystals present in the as-synthesized film and created during film formation are partially melted and act as epitaxial nuclei in the subsequent cooling scans. A final heating scan at 20 °C/min is performed to observe the melting of the crystals formed (Figures S6b, S7b, S8b, S9b, Supporting Information).

The F.A1 and F.A2 samples based on the GDMA–DATP monomer pair display similar behavior. In the cooling scans from the range of selected *T*_s_ (Figures S6a and S7a, Supporting Information), *T*_c_ does not significantly change with *T*_s_ for F.A1 (Figure 6b) and slightly increases at lower *T*_s_ for F.A2 (Figure 6c). In the following DSC heating scans, traces of annealing can be appreciated with clear multimodal peaks shown in Figures S6b and S7b.

Hence, polymers based on GDMA–DATP are in the SN Domain III in the selected *T*_s_ range. When *T*_s_ values were increased above 57 °C, no recrystallization peak could be observed by DSC, showing that the GDMA–DATP polymer cannot crystallize when in *Domain II* and above. The absence of *Domain II*, or the self-nucleation domain, the GDMA–DATP polymer does not display a melt memory effect or even a self-seeding effect without annealing.^23^

In the DSC heating scans shown in Figures S8b and S9b in the Supporting Information, traces of annealing can be appreciated for the F.B2 film at *T*_s_ lower than 58 °C and for the F.C film at *T*_s_ lower than 66 °C showing that the films are in *Domain III* below these temperatures. At higher *T*_s_, F.B2 and F.C are in *Domain II*, with a clear increase of *T*_c_ as *T*_s_ values decreased, as seen in Figure 6d,e.

Table 1 and Figure 6 summarize the characteristics of each film in terms of chemical structure, molar mass, *T*_g_, and number of domains. It has been reported that when studying the melt-memory effect by SN experiments, the effect of chemical structure can be investigated by comparing the width of the self-nucleation domain (or *Domain IIb*).^35^ In view of the results obtained for the studied films, we propose here a comparison in terms of the number of SN domains. As highlighted in Figure 6, the F.B1.HD film is the only one displaying the three SN domains. As supported by the previous non-isothermal study, F.B1.HD exhibits faster crystallization kinetics, possibly caused by a low *T*_g_ of −22.6 °C and the presence of HD. When compared to its counterpart F.B2, which displays the same chemical structure and nearly the same *T*_g_ but only two SN domains, faster crystallization of F.B1.HD can be explained by the lower molar mass and the presence of HD.

The F.C film also exhibits two SN domains, highlighting its slower crystallization kinetics compared to F.B1.HD. The presence of an additional ester group within the polymer repeating unit, coming from the GDMP monomer (see Figure 1 and Table 1), may enhance chain rigidity in comparison to F.B1.HD and F.B2, causing a higher *T*_g_ (−14.6 °C) value and slowing down the crystallization kinetics compared to F.B1.HD and F.B2.

Finally, the SN experiments reveal that the films based on GDMA–DATP polymer (F.A1 and F.A2) present the slowest crystallization kinetics with crystallization occurring only in *Domain III*. The slowest crystallization kinetics of GDMA–DATP films is due to their more rigid chemical structure, which on one hand, has the aromatic groups and, on the other, a higher density of ester groups than in the GDMA-based polymer.

To gain deeper insight into poly(thioether) crystalline behavior, the degree of crystallinity *X*_c_ at each *T*_s_ was calculated for the studied films using eq 2, and the results are shown in Figure S10, Supporting Information. Compared with *X*_c_ calculated for the as-synthesized samples presented in Table 1, the degree of crystallinity after SN experiments slightly decreases for the F.A1, F.B1.HD, F.B2, and F.C samples Even though the % crystallinity present in the as-synthesized samples was not fully recovered, a much faster crystallization from the molten state was achieved for these polymers using the SN strategy schematized in Figure 2b. Notably, the degree of crystallinity after SN was increased in the case of the F.A2 film at *T*_s_ values of 45 °C, 46 °C, and 47 °C with *X*_c_ values above 16%.

**Figure 7:**
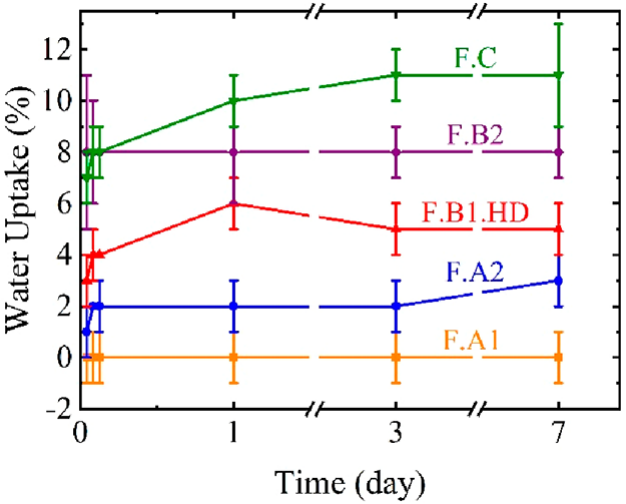
Water uptake for F.A1, F.A2, F.B1.HD, F.B2, and F.C film samples.

The water sensitivity of polymer films intended for coating applications is an important characteristic and is determined by water uptake measurements. For that aim, the water uptake of the F.A1, F.A2, F.B1.HD, F.B2, and F.C films were measured during 7 days; the results are shown in Figure 7.

The water absorption of waterborne films is often attributed to the migration of the hydrophilic surfactant during the film formation process, which creates aggregates within the film able to absorb large quantities of water from the surroundings. The presence of crystalline structures within the present films might limit water diffusion by forming physical obstacles within the film. Indeed, in the case of PVDC, excellent barrier properties are provided by the presence of crystalline structures with a degree of crystallinity *X*_c_ ranging from 35% to 50%.^28,29^

The studied films present low water uptake after 1 day (below 12%), and noticeably the F.A1 film does not absorb any water with a water uptake close to 0%. This is a very unusual result for waterborne polymer films synthesized using conventional surfactants (Dowfax 2A1 in this case). As the three films present a similar degree of crystallinity of ∼20%, no link between the crystallinity and the water diffusion could be established. The difference in water uptake is attributed to the film quality obtained after water evaporation. The low *T*_g_ of the polymers in the −7.8 °C to −22.6 °C range allows the formation of very good quality films for all polymers studied. The crystals developed during film formation, and the aromatic structure of the polymers probably provided the necessary stiffness. In addition, F.A1 presents the lowest molar mass of all studied films, which probably positively affected the film formation process. It is well-known that the interdiffusion of the lower molar mass polymer chains between the borders of polymer particles is easier.^25^ Therefore, due to its low molar mass of about 9 kDa, the interdiffusion of the polymer chains was facilitated, which, along with the low *T*_g_, resulted in a very compact film. As only the FA1 film was obtained without an initiator, the lack of hydrophilic moieties in the chains increased their hydrophobicity. Finally, the crystal aggregates which formed inside the films probably decreased even more the water permeability.

Water vapor permeation tests were performed on the F.A1 and F.C films, and results are presented in Table 1. The F.B1.HD and F.B2 films were too brittle and thus could not be tested.

In line with the data obtained by water uptake experiments, the F.A1 film presents the best results in terms of water vapor transfer. Moreover, the obtained value is within the range of water vapor permeation data reported for commonly used commercial barrier coatings, which are between 0.1 and 30 (g·mm)/(m^2^·day).^1^ This means that the crystalline structures are distributed within the amorphous matrix in a way to create a barrier to the paths that might be created by the hydrophilic species present in the film (initiator and surfactant). This result unlocks the potential of the waterborne poly(thioethers) in barrier coating applications as a possible more environmentally friendly replacement for the current PVDC coatings.

## Conclusions

We have performed successful synthesis of waterborne film-forming poly(thioether) latexes by sonopolymerization combined with photopolymerization, based on the DATP diene monomer and various dithiol monomers, such as GDMA, GDMP, and EDDT. Waterborne coatings based on thiol–ene chemistry were prepared, and their crystallization behavior was studied for the first time.

As the selected monomers are bifunctional, the thiol–ene step growth mechanism facilitates the synthesis of linear poly(thioethers) with semicrystalline behavior evidenced by DSC analysis. The as-synthesized films present a degree of crystallinity of approximately 20% and slow crystallizing polymer chains. The SN thermal protocol turns out to be an efficient strategy to study the crystallization behavior of such polymers, as the produced self-nuclei substantially accelerate the overall crystallization kinetics. This study allowed correlation of the chemical structure of the poly(thioethers) with their non-isothermal crystallization, by comparing the number of SN domains that can be obtained through the technique. Faster non-isothermal overall crystallization kinetics were obtained for the coatings containing HD and based on the EDDT–DATP poly(thioether) chains, devoid of pendant moieties, and with low *T*_g_ values. The presence of HD could act as a plasticizer and promote the faster crystallization of polymer chains.

All films presented low water uptake after 1 week (below 12%), and the best results were obtained for the film based on GDMA–DATP sonopolymers. These also exhibited the lowest WVTR (2 (g·mm)/(m^2^·day)). Noteworthy, this coating does not contain the water-soluble photoinitiator (used in all other formulations) and contains shorter polymer chains, which could more efficiently interdiffuse during the film formation process yielding a homogeneous film after water evaporation. The films obtained from EDDT–DATP and GDMP–DATP presented some inhomogeneities, which could explain their higher water uptake. As the values of the films are not significantly different (ranging from 15 to 25%), no link between *X*_c_ and water uptake could be concluded.

## Supporting Information Available

Back-light scattering graphs showing minimeulsion stability; photos of the polymer films after drying; TGA graphs of the polymer films; non-isothermal DSC analysis of HD; graphs of the self-nucleation experiments for poly(thioether) films (PDF)
