# Supplementary material for: Synthesis and Crystallization of Waterborne Thiol–ene Polymers: Toward Innovative Oxygen Barrier Coatings
Source: ACS Appl Polym Mater. 2023 Oct 20;5(11):8845–58. doi: 10.1021/acsapm.3c01128 (PMC10644330; doi:10.1021/acsapm.3c01128)
Supplement: Supplementary file 1 — ap3c01128_si_001.pdf [file ap3c01128_si_001.pdf]

Supporting information for Synthesis and crystallization of waterborne thiol-ene polymers: towards innovative oxygen barrier coatings.

Justine Elgoyhen,[a] Valentina Pirela,[b] Alejandro J. Müller,[b,c,*] Radmila Tomovska[a,c,*].

[a] POLYMAT and Department of Applied Chemistry, Faculty of Chemistry, University of the Basque Country UPV/EHU, Avda Tolosa 72, 20018 Donostia-San Sebastián, Spain

[b] POLYMAT and Department of Polymers and Advanced Materials: Physics, Chemistry and Technology, Faculty of Chemistry, University of the Basque Country UPV/EHU, Paseo Manuel de Lardizábal, 3, 20018 Donostia-San Sebastián, Spain

[c] IKERBASQUE, Basque Foundation for Science, Plaza Euskadi 5, 48009, Bilbao, Spain

Table S1. Formulations and synthesis conditions of 30 wt% and 50 wt% solids content sonopolymers or prepolymers obtained by sonopolymerization of the bifunctional monomers DATP, GDMA, EDDT and GDMP.

Entry	Monomers	Mass DATP (g)	Mass Thiol (g)	AIBN (wt%)*	HD (wt%)*	Solids content (%)	Sonication time (min)
**S.1**	GDMA-DATP	22.4483	19.7849	-	-	30	10
**S.2**	GDMA-DATP	22.4463	19.7848	-	-	30	10
**S.3**	GDMA-DATP	22.4488	19.7746	0.2	-	30	10
**S.4**	EDDT-DATP	19.3880	14.8058	-	6	30	7.5
**S.5**	EDDT-DATP	19.3893	14.8128	-	-	30	7.5
**S.6**	GDMP-DATP	19.3995	19.3857	-	-	30	7.5
**S.7**	EDDT-DATP	32.6515	24.9355	-	-	50	10
**S.8**	EDDT-DATP	32.6533	24.9556	-	-	50	12
**S.9**	GDMA-DATP	37.7575	33.2591	-	-	50	20
**S.10**	GDMP-DATP	32.6453	32.3754	-	-	50	10

*Based on monomers' mass

Table S2. Calculated $\Delta H_{m,100\%\ crystalline}$ with the group contribution theory for the films based on the polymers GDMA-DATP, GDMP-DATP, and EDDT-DATP monomer pairs.

Film	Monomers	$\Delta H_{m,100\%\ crystalline}$ (J/g)
**F.A1, F.A2**	GDMA-DATP	108
**F.B1.HD, F.B2**	EDDT-DATP	151
**F.C**	GDMP-DATP	119

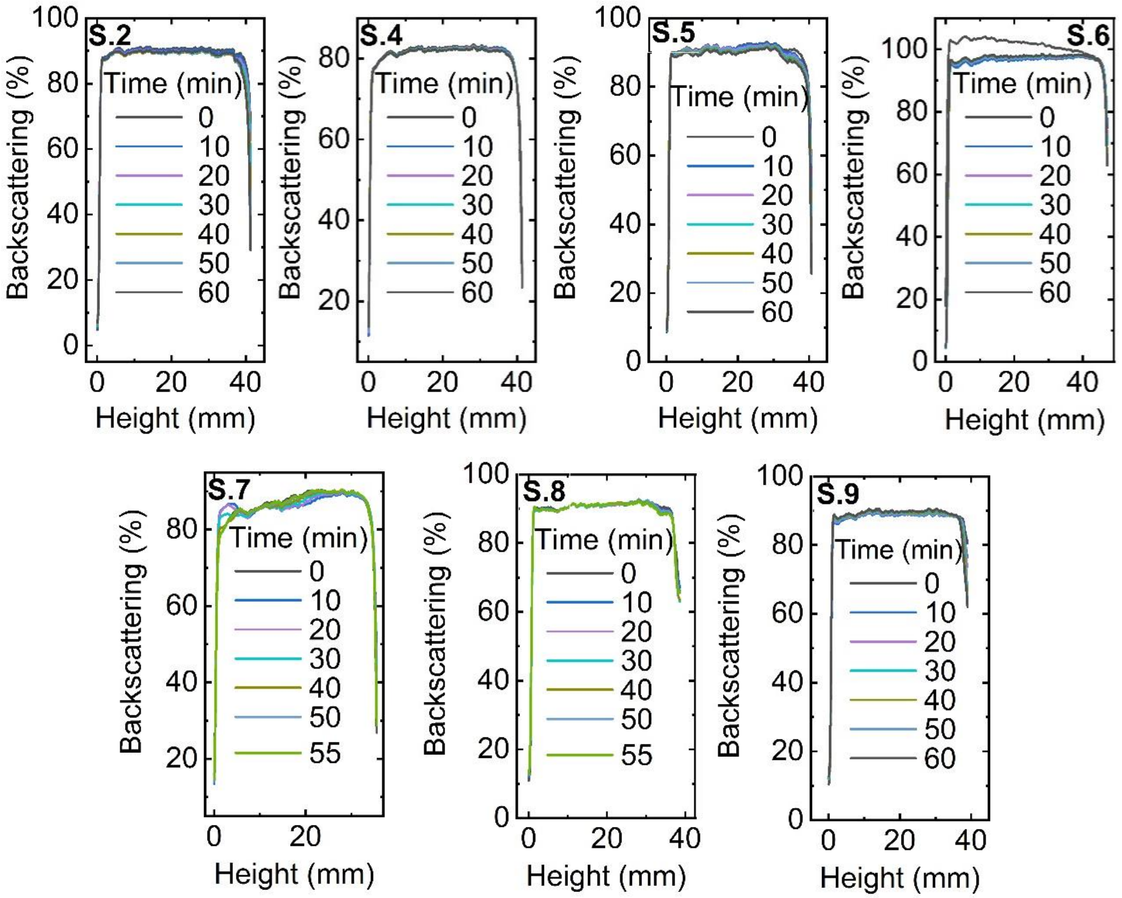

Figure S1. Colloidal stability of 30% and 50% solids content prepolymer latexes S.2, S.4, S.5, S.6, S.7, S.8 and S.9

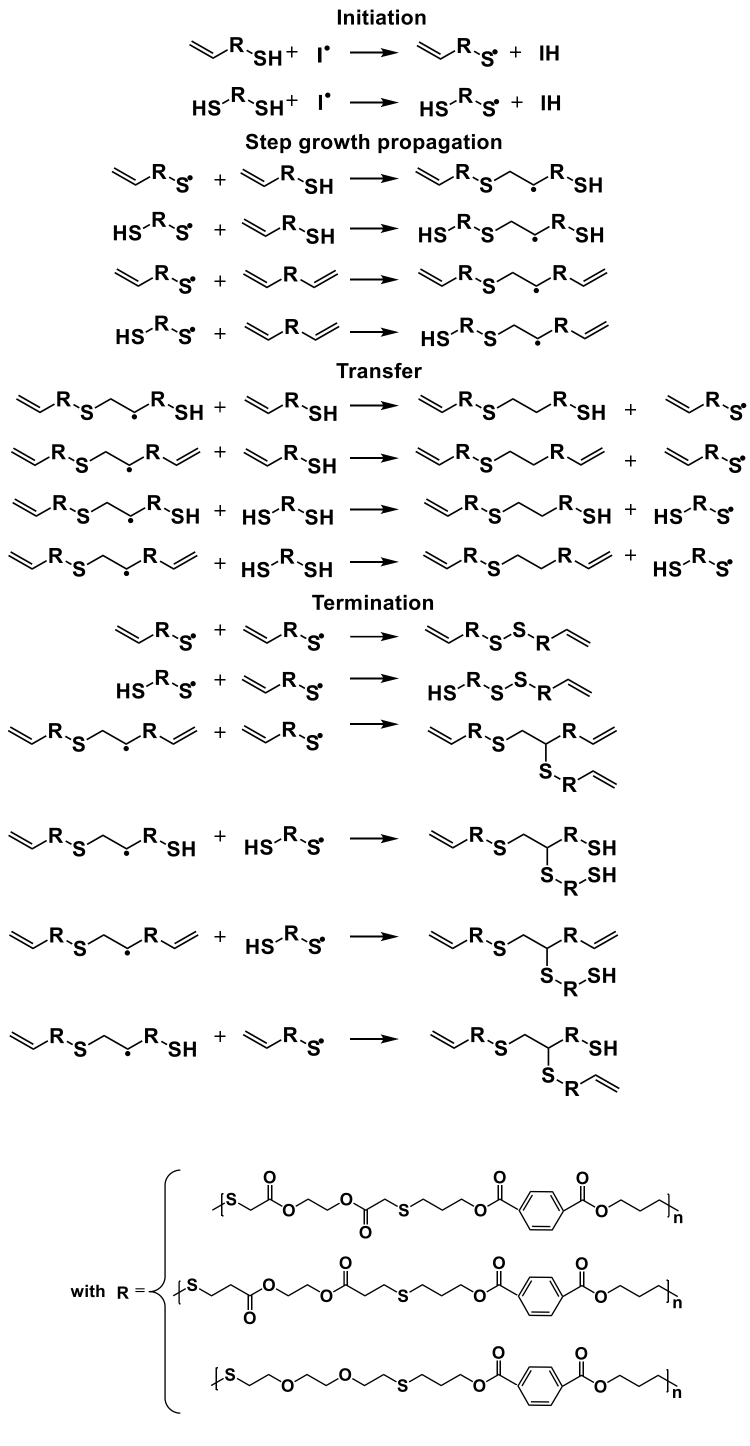

Figure S2. Photopolymerization mechanism of the thiol-containing prepolymers. R stands for a moiety that contains i-number of thiol-ene monomer units (i > 1) in the oligoradicals or prepolymer chains.

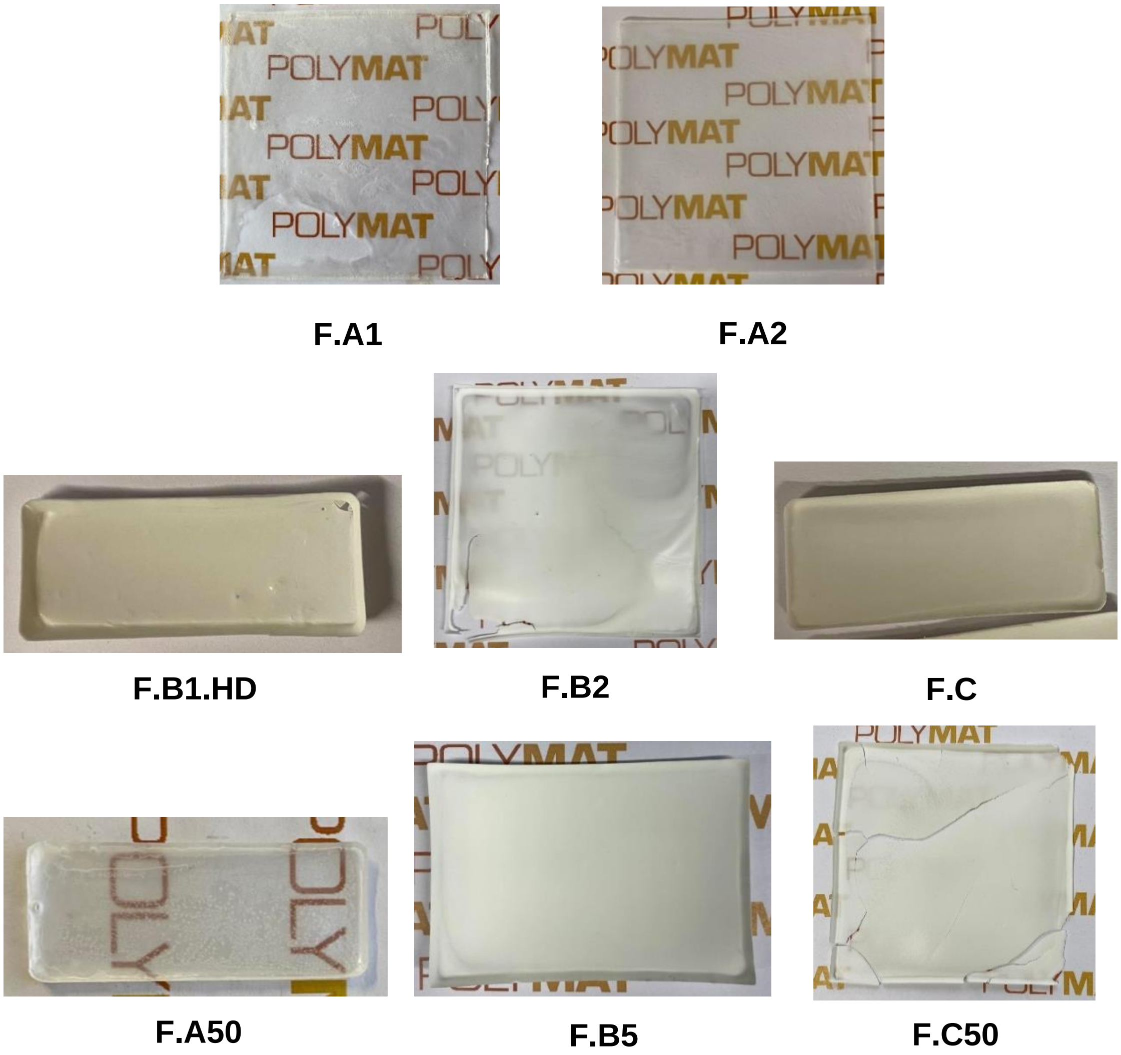

Figure S3. Films F.A1, F.A2, F.B1.HD, F.B2 and F.C obtained after water evaporation of the 30% solids content latexes and films F.A50, F.B50 and F.C50 obtained after water evaporation of the 50% solids content laxetes based on GDMA-DATP, GDMP-DATP and EDDT-DATP. Films were casted into silicon mold and dried at controlled temperature (25°C) and humidity (55%).

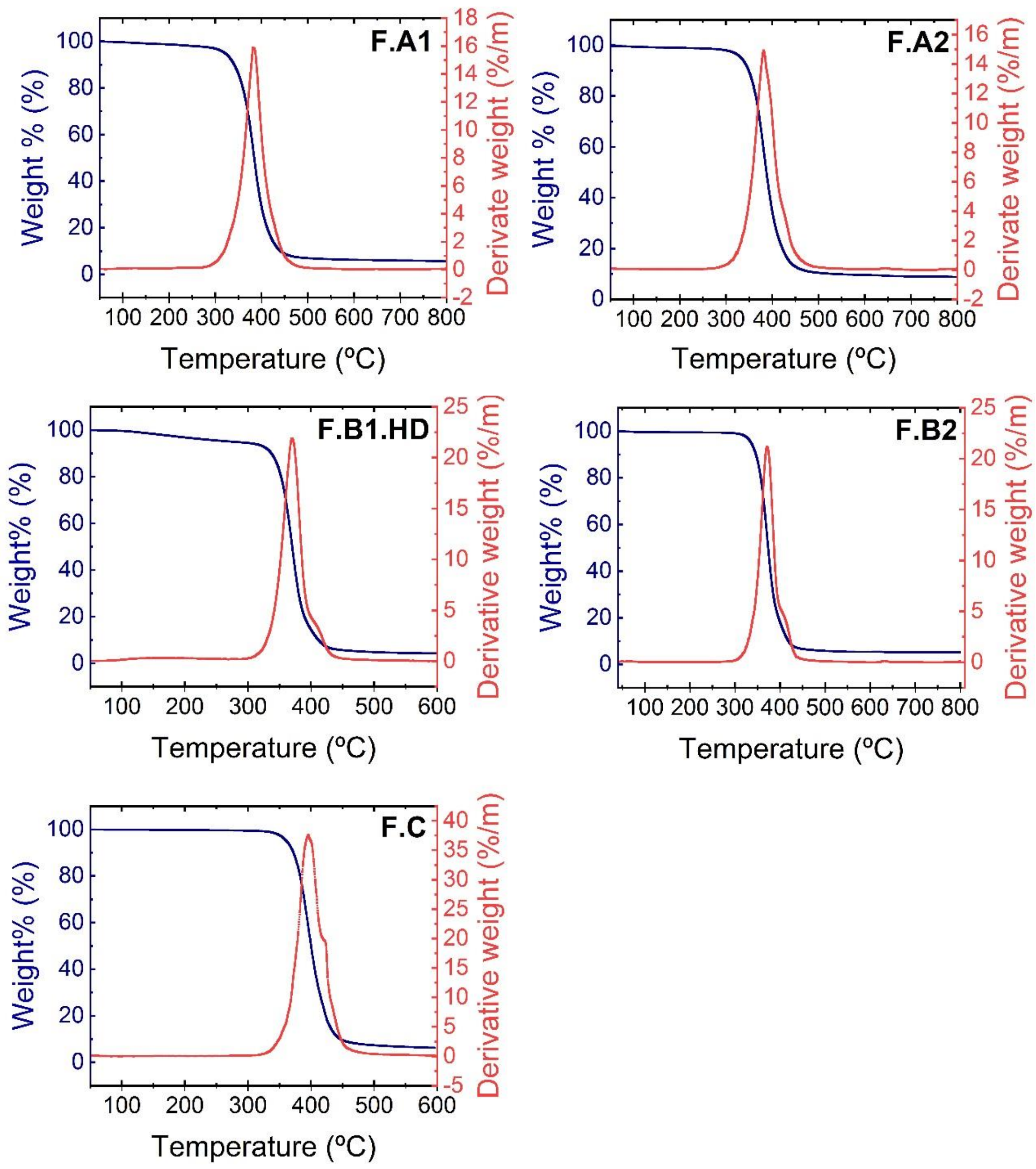

Figure S4. TGA of the films F.A1, F.A2, F.B1.HD, F.B2, and F.C.

Table S3. Melting temperatures (T_m) and melting enthalpy (ΔH_m) values from the first heating scan. Cold crystallization enthalpy (ΔH_{cc}), T_m, ΔH_m, and glass transition temperature (T_g) values from the second heating scan following a cooling ramp from the molten state at 1 ºC/min. Values are extracted from the non-isothermal experiments performed on F.A1, F.A2, F.B1.HD, F.B2 and F.C reported in Figure 3..

			**First heating scan**		**Second heating scan**			
**Film**	**Monomers**	**HD (wt%)**	**T_m (ºC)**	**ΔH_m (J/g)**	**ΔH_{cc} (J/g)**	**T_m (ºC)**	**ΔH_m (J/g)**	**T_g (ºC)**
**F.A1**	GDMA-DATP	-	42.5/49.1/55.6	23	-	-	-	-15.6
**F.A2**	GDMA-DATP	-	45.1/52.5/57.4	16	-	-	-	-7.8
**F.B1.HD**	EDDT-DATP	6	48.2/61.7	37	5	25.3/60.9	27	-22.3
**F.B2**	EDDT-DATP	-	42.5/61.6	33	-	61.3	15	-22.6
**F.C**	GDMP-DATP	-	45.1/67.0	23	-	-	-	-14.6

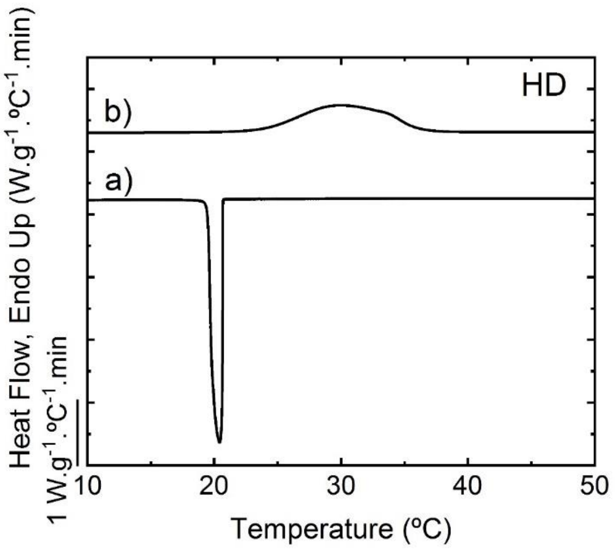

Figure S5. Non isothermal DSC analysis of HD a) DSC cooling scan from molten state at 1ºC/min and b) subsequent heating scan at 20ºC/min.

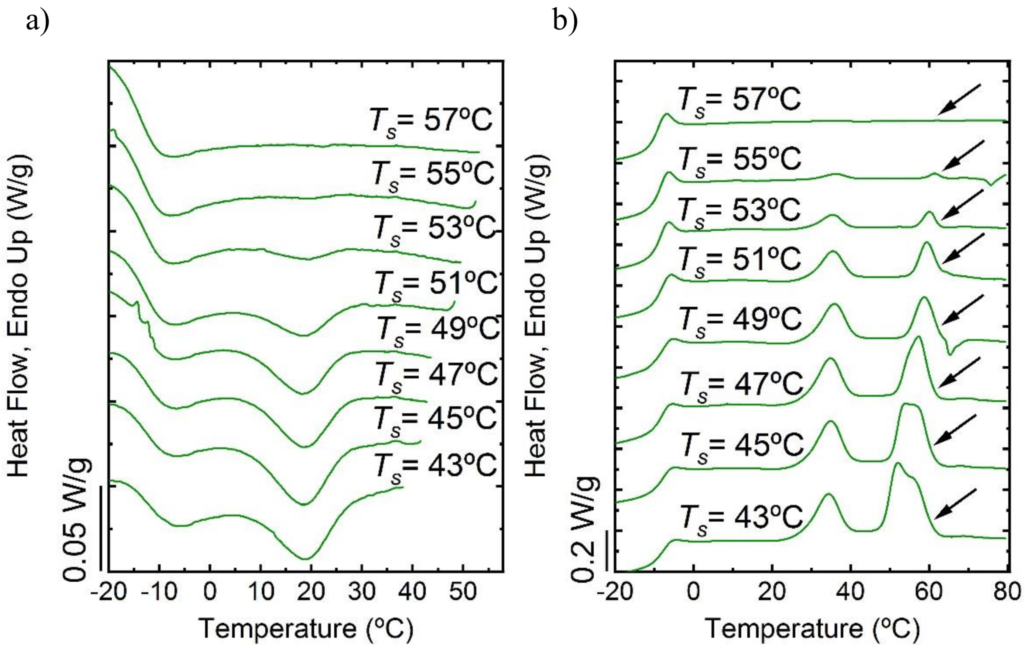

Figure S6. Self-nucleation experiments for poly(thioether) film F.A1: a) DSC cooling scans from the indicated T_s temperatures at 10 ºC/min and b) subsequent heating scans at 20 ºC/min. Arrows show traces of annealing.

a) b)

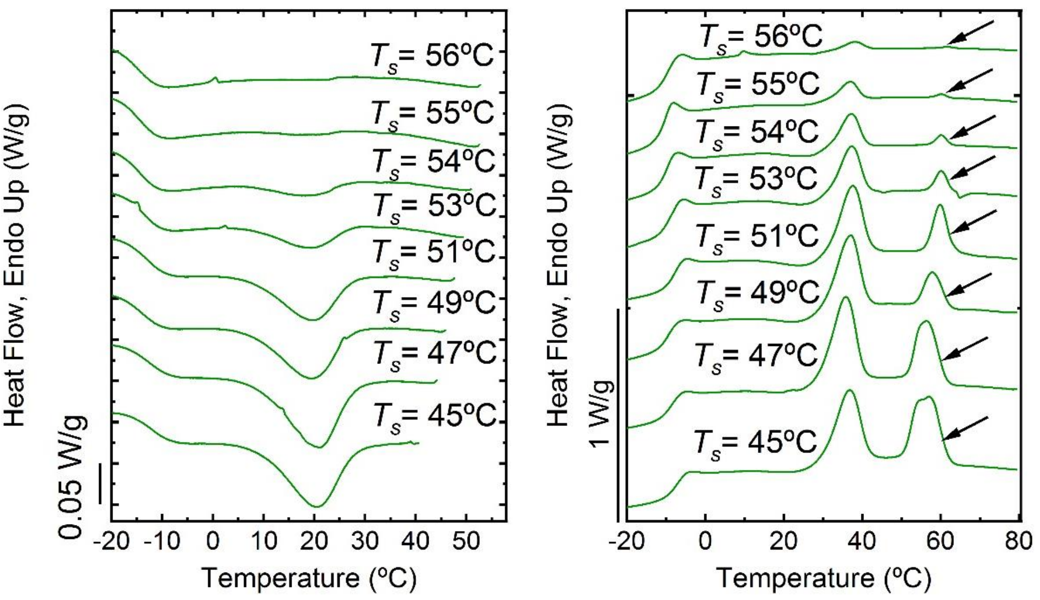

Figure S7. Self-nucleated poly(thioether) film F.A2: a) DSC cooling scan from the indicated T_s temperatures at 10 ºC/min and b) subsequent heating scans at 20 ºC/min. Arrows show traces of annealing

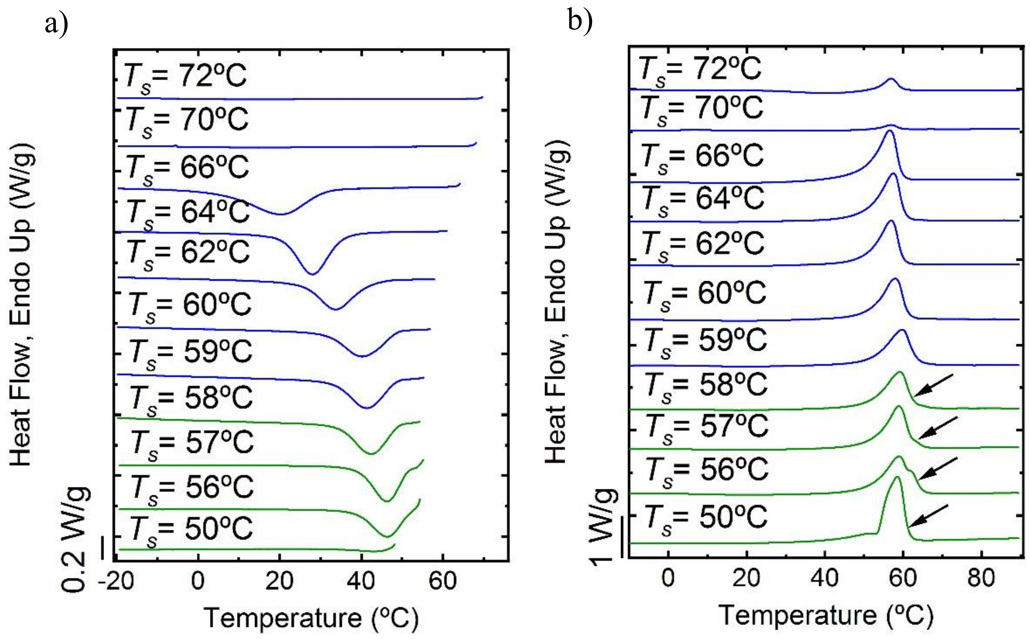

Figure S8. Self-nucleation experiments for poly(thioether) film F.B2: a) DSC cooling scans from the indicated T_s temperatures at 10 ºC/min and b) subsequent heating scans at 20 ºC/min. Arrows show traces of annealing.

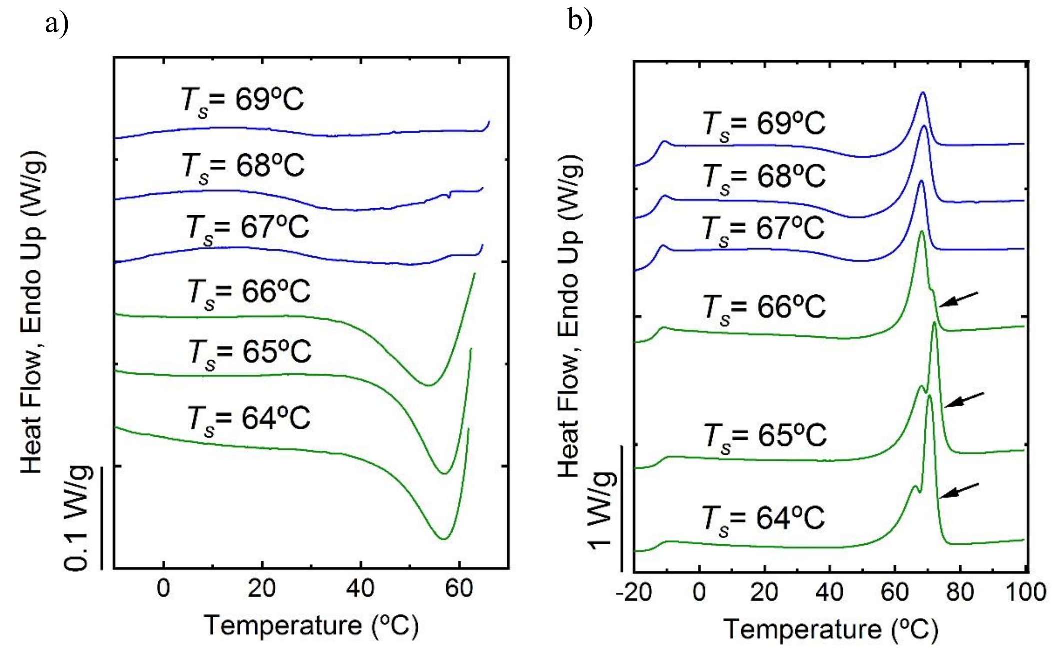

Figure S9. Self-nucleation experiments for poly(thioether) film F.C: a) DSC cooling scan from the indicated T_s temperatures at 10 ºC/min and b) subsequent heating scans at 20 ºC/min. Arrows show traces of annealing.

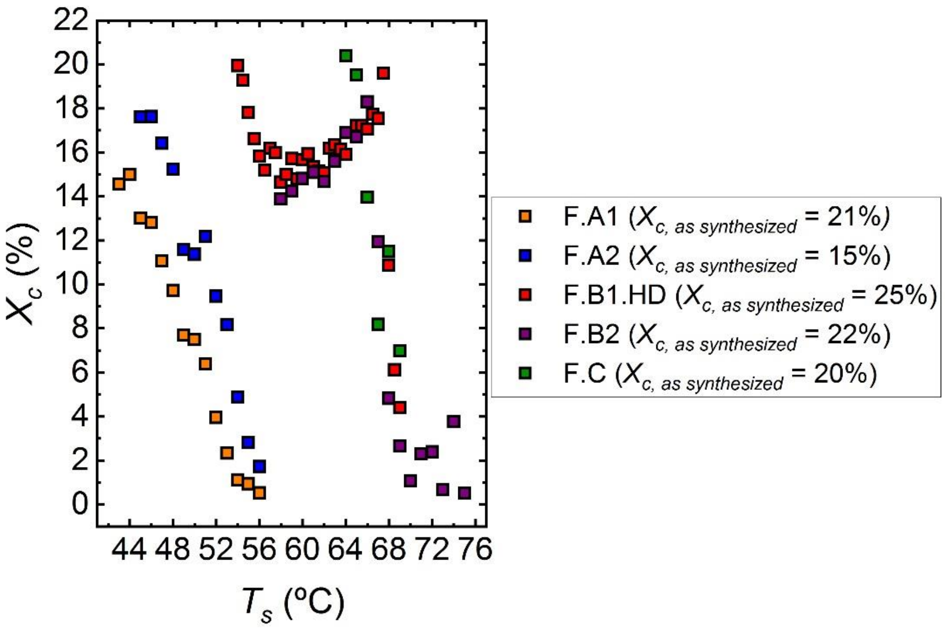

Figure S10. Degree of crystallinity (X_c) against T_s for the F.A1, F.A2, F.B1.HD, F.B2 and F.C films.
